# Mislocalization of PBPs in *Staphylococcus aureus gdpP* mutant contributes to β-lactam resistance and surface protein cross-wall trafficking

**DOI:** 10.1128/spectrum.01430-26

**Published:** 2026-08-12

**Authors:** Yaosheng Jia, Ran Zhang, Salvatore J. Scaffidi, Wenqi Yu

**Affiliations:** 1 Department of Molecular Biosciences, College of Arts and Sciences, University of South Florida 122476, Tampa, Florida, USA; 2 Center for Antimicrobial Resistance, University of South Florida 7831, Tampa, Florida, USA; Texas A&M University, College Station, Texas, USA

**Keywords:** *Staphylococcus aureus*, GdpP, β-lactam resistance, PBP1, PBP2, PBP3, PBP4, YSIRK/G-S protein, SpA

## Abstract

GdpP is a phosphodiesterase that degrades the second messenger c-di-AMP, which plays a key role in osmoregulation and antibiotic resistance in many gram-positive bacteria. Mutations in *gdpP* are frequently associated with *mecA*-independent β-lactam resistance in *Staphylococcus aureus*, but the mechanisms remain unknown. Here, we show that deletion of *gdpP* diminished cross-wall deposition of YSIRK+ surface protein A (SpA). Strikingly, all four native PBPs (PBP1–4) were mislocalized as distinct single foci in Δ*gdpP* cells. Deficiency in cell division initiation accumulated non-dividing cells in Δ*gdpP*, and the aberrant PBP foci were predominantly found at the cell periphery of non-dividing cells. The mislocalization of PBPs is due to the accumulation of c-di-AMP, as shown in both Δ*gdpP* and *dacA* (c-di-AMP synthase) overexpression strains. The mislocalized PBPs were largely inactive as they were unable to incorporate FDAAs locally. Δ*gdpP* did not show significant change in overall peptidoglycan cross-linking. Consistently, Δ*gdpP* strains were resistant to β-lactams that target PBPs, but not to vancomycin that targets peptidoglycan cross-linking. Finally, the aberrant PBP foci formation correlates with resistance to β-lactams-mediated killing, but not to vancomycin. Based on these results, we propose that increased c-di-AMP level in Δ*gdpP* cells leads to aggregation of PBPs into peripheral foci that mis-target β-lactams and delay in cell division dysregulates YSIRK+ protein cross-wall deposition. Our results provide new mechanistic insight into connections among c-di-AMP signaling, β-lactam resistance, and YSIRK+ virulence protein assembly that are widely found in gram-positive bacteria.

**IMPORTANCE:**

C-di-AMP signaling pathway is widely found in bacteria and frequently associated with antibiotic resistance and tolerance. In *Staphylococcus aureus*, mutations in *gdpP* encoding the phosphodiesterase that degrades c-di-AMP have been associated with *mecA*-independent β-lactam resistance, while the mechanisms are largely unknown. Here, we report new findings that accumulation of c-di-AMP in the *gdpP* mutant leads to aberrant foci formation of all four native PBPs in *S. aureus*, which correlates with resistance to β-lactam antibiotics. Moreover, the *gdpP* mutant is deficient in cell division, which diminishes cross-wall assembly of YSIRK+ surface virulence proteins. Our study provides new insight into connections among c-di-AMP signaling, β-lactam resistance, and YSIRK+ virulence protein biogenesis that are widely found in gram-positive bacteria, and lays a foundation for developing new therapeutics.

## INTRODUCTION

*Staphylococcus aureus* is a gram-positive bacterium that frequently colonizes human nares and skin (1). It is also one of the most important pathogenic bacteria, responsible for causing various infections, such as skin and soft tissue infections, pneumonia, endocarditis, osteomyelitis, and life-threatening sepsis (2–4). β-Lactam antibiotics, including cephalosporin and oxacillin, are frequently used for *S. aureus* infections in clinical settings (5, 6). However, the rise of antibiotic-resistant *S. aureus*, including community and hospital MRSA (methicillin-resistant *S. aureus*), poses a significant challenge to these primary treatment methods (7, 8). β-Lactam antibiotics inhibit the transpeptidase activity of penicillin-binding proteins (PBPs), which is essential for cell-wall peptidoglycan (PG) cross-linking. Remarkably, *S. aureus* has evolved various resistance mechanisms against generations of β-lactam antibiotics over the years. β-Lactamase encoded by BlaZ contributes to narrow-spectrum resistance by degrading β-lactam antibiotics (9). The presence of *mecA* gene, which encodes PBP2a, renders resistance to β-lactamase-resistant antibiotics in MRSA strains (10, 11). Recent studies of *mecA*-negative MRSA strains reveal that mutations in the *gdpP* gene are frequently associated with β-lactam resistance and tolerance in both clinical and laboratory isolates (12–25).

GdpP (GGDEF domain protein containing phosphodiesterase) in *S. aureus* is a close homolog to YybT in *Bacillus subtilis* and Pde1 in *Streptococcus pneumoniae*, which is known to hydrolyze c-di-AMP, a second messenger widely found in bacteria (13, 26, 27). In *S. aureus*, a gene named *dacA* encodes for a diadenylate cyclase that synthesizes c-di-AMP from two ATP molecules (28). While the association of increased c-di-AMP with decreased β-lactam susceptibility has been reported in several gram-positive bacteria, including *S. aureus*, *Bacillus subtilis*, *Lactococcus lactis*, *Listeria monocytogenes*, *Streptococcus pyogenes*, *Streptococcus agalactiae*, and *Enterococcus faecalis* (13, 29–38), the underlying mechanisms are largely unknown. C-di-AMP regulates multiple facets of bacterial physiology, such as cell volume, osmotic homeostasis, cell wall homeostasis, metabolism, DNA repair, and virulence (13, 39–44). One of the conserved functions of c-di-AMP across bacterial species is osmoregulation by binding to proteins and riboswitches that regulate potassium and compatible solutes transportation (45, 46). In *S. aureus*, c-di-AMP has been shown to bind to KtrA (potassium transporter-gating component), CpaA (a predicted cation/proton antiporter), PstA (PII-like signal transduction protein A), KdpD (a sensor histidine kinase that activates the expression of potassium transporter operon), and OpuCA (a component of the osmolyte uptake system) (47, 48). Either *gdpP* mutation or *dacA* overexpression leads to c-di-AMP accumulation, which negatively influences potassium and other osmolytes uptake; decreased potassium uptake further leads to cell shrinkage, decreased turgor pressure, and smaller cell size (13, 14, 22, 49–51). A recent study showed that the levels of potassium and free amino acids correlate with antibiotic sensitivity in *L. lactis* (34). It is hypothesized that an increased c-di-AMP level reduces cellular turgor pressure, generating resistance to osmotic stress and β-lactam antibiotics (34, 46). However, *S. aureus* Δ*gdpP* did not show resistance toward other cell wall-targeting antibiotics, such as moenomycin and bacitracin (22), suggesting that the resistance mechanism may not be limited to the turgor pressure. Another possible explanation is that elevated c-di-AMP alters cell wall structure by increasing cell wall cross-linking and thickness (13, 22, 23). However, the results are inconsistent in the literature in terms of cell wall cross-linking: Δ*gdpP* showed higher cross-linking in a previous study but no change in PG profile as reported in a more recent study (13, 23). Furthermore, c-di-AMP homeostasis has been connected to PG precursor synthesis (36, 52, 53); however, the mechanisms between PG precursor synthesis and β-lactam resistance remain underexplored.

In addition to conferring antibiotic resistance, mutation of *gdpP* can suppress the lethality of *S. aureus* Δ*ltaS*, deficient in lipoteichoic acid (LTA) production (13). LTA is a major cell envelope component of gram-positive bacteria, and loss of LTA leads to severe cell growth retardation, cell division defect, and cell envelope stress (54, 55). In our previous study, we investigated the role of Δ*ltaS* and Δ*ltaS*Δ*gdpP* in regulating cell wall-anchored YSIRK/G-S surface proteins (56). Surface proteins are a group of proteins that are covalently attached to cell wall PG, which are essential virulence factors in *S. aureus* (57). The precursors of surface proteins are secreted across the membrane through the Sec translocon and covalently attached to PG precursor lipid II by the membrane-bound transpeptidase sortase A (58–62). Lipid II-linked surface protein precursors are incorporated into mature peptidoglycan via PBP/SEDS-mediated transpeptidation and transglycosylation during cell wall synthesis (58). Specifically, surface proteins carrying a YSIRK/G-S signal peptide (YSIRK+ proteins) are enriched at the cell division septum and anchored to the septal peptidoglycan (cross-wall) (56, 63). Thus, the dynamic localization of YSIRK+ proteins is coupled with the cell cycle and cell wall biosynthesis. We found that the septal trafficking of staphylococcal surface protein A (SpA), an archetype of YSIRK+ proteins and a key virulence factor in *S. aureus* immune evasion, was disrupted in strain ANG1786 (Δ*ltaS*Δ*gdpP*) (56). Interestingly, complementation with *ltaS* only restored SpA septal localization, but not its cross-wall localization (data not shown), suggesting that *gdpP* mutation alone may play a role in regulating SpA cross-wall targeting.

In this study, we investigated the role of *gdpP* single mutant in SpA localization, PBPs’ localization, and β-lactam susceptibility. We found that *gdpP* mutation diminished SpA cross-wall deposition and triggered aberrant foci formation of all four PBPs in *S. aureus*. The rate of PBP foci formation correlated with the level of intracellular c-di-AMP accumulation. The PBPs were predominantly found in non-dividing cells, and the PBPs in the foci were mostly inactive. The overall PG cross-linking was not significantly changed in Δ*gdpP*. The Δ*gdpP* strains were resistant to PBP-targeting β-lactams but not to vancomycin, which targets PG cross-linking. Consistently, the foci formation of PBPs was resistant to β-lactams but not to vancomycin. We propose that increased c-di-AMP in Δ*gdpP* cells leads to mislocalization of PBPs and cell cycle defects, which are the underlying mechanisms connecting c-di-AMP signaling, β-lactam resistance, and YSIRK+ protein spatial regulation.

## RESULTS

### Δ*gdpP* diminishes SpA cross-wall deposition in a YSIRK/G-S signal peptide-dependent manner

As previously noted, SpA cross-wall localization remained defective in Δ*ltaS*Δ*gdpP* despite *ltaS* complementation. To further investigate this, we generated a Δ*gdpP::erm* internal deletion mutant in strain SEJ1 (a *spa* deletion mutant of RN4220) (Fig. S1). The *gdpP* gene is located adjacent to two essential genes in a four-gene operon. To avoid potential polar effects, the *erm* marker was removed via the *cre-lox* system, generating a markerless *gdpP* deletion mutant (64). The *spa* gene is expressed from its native promoter in pCL55 integrated at an ectopic locus (56).

**Fig 1:**
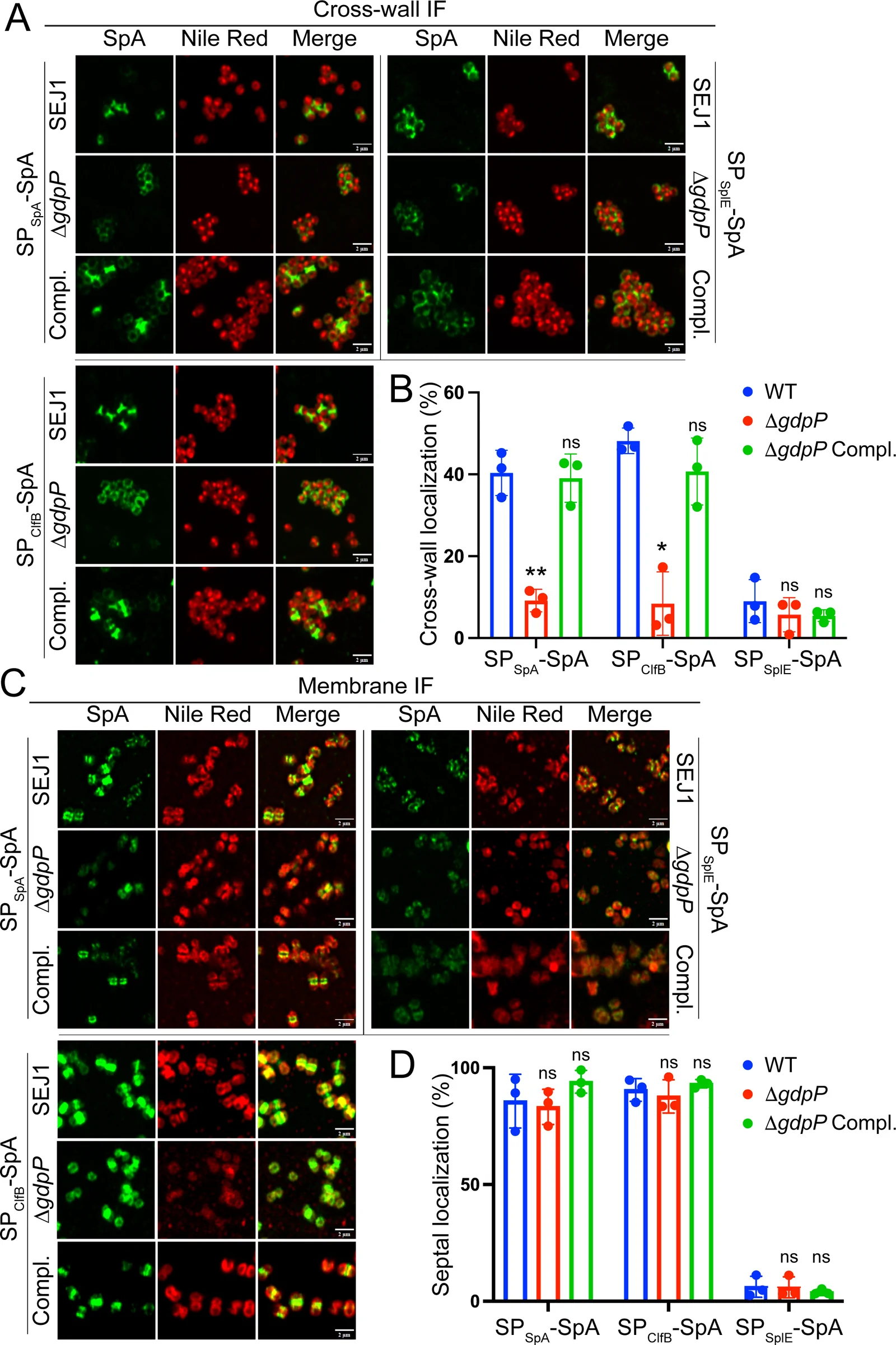
Δ*gdpP* diminishes the cross-wall deposition of SP_SpA_-SpA, SP_ClfB_-SpA, but not SP_SplE_-SpA. SP_SpA_ and SP_ClfB_ are YSIRK+ signal peptides, and SP_SplE_ is a YSIRK- signal peptide. (**A**) Cross-wall IF images showing localization of SpA fused with different signal peptides in *S. aureus* SEJ1 WT, Δ*gdpP*, and *gdpP* complementation strains. SP-SpA fusions are expressed from *spa* native promoter in pCL55. EV, empty vector of pCL55. Nile red stains cytoplasmic membrane. Scale bar, 2 µm. (**B**) Quantification of SpA cross-wall localization from panel **A**. (**C**) Membrane IF images showing membrane-proximal localization of SpA fused with different signal peptides. (**D**) Quantification of SpA septal membrane localization. Unpaired *t*-test with Welch’s correction was performed for statistical analysis: ns, not significant; *P < 0.05; **P < 0.005. Representative images and quantification are from three independent experiments.

To examine the subcellular localization of SpA, we used two immunofluorescence (IF) methods that we established previously: cross-wall IF and membrane IF (65–67). In a cross-wall IF experiment, staphylococcal cells are treated with trypsin to remove the pre-existing SpA and further incubated in fresh medium for 20 min, allowing the deposition of newly synthesized SpA. Thus, cross-wall IF reveals the localization of surface-exposed and cell wall-anchored SpA. As shown in Fig. 1, SpA localized at the cross-wall in wild-type (WT) cells. The cross-wall localization of SpA was strongly reduced, and the peripheral SpA signals were increased in SEJ1Δ*gdpP* cells (Fig. 1A and B, SP_SpA_-SpA). SpA fluorescence signal intensity was also reduced in Δ*gdpP*. Complementation by plasmid-borne *gdpP* expression restored the cross-wall localization of SpA. A second experiment is membrane IF, which examines SpA localization proximally to the membrane beneath the PG layer. Briefly, trypsinized cells are further treated with cell wall hydrolase lysostaphin, which removes the PG layer and separates the dividing daughter cells, enabling SpA antibody to access the septal membrane. SpA localizes at the septal membrane in WT cells, and its localization was not significantly altered in Δ*gdpP,* as revealed by microscope imaging and quantification (Fig. 1C and D). To examine if the phenotype is conserved in *S. aureus*, we transduced Δ*gdpP::erm* to MSSA strains Newman and HG001 and MRSA strain USA300 JE2. The same results were observed in different strain backgrounds: Δ*gdpP* diminished SpA cross-wall localization but not its septal membrane localization (Fig. S2).

To investigate whether the phenotype is dependent on the YSIRK/G-S signal peptide, the signal peptide of SpA was replaced with another YSIRK+ signal peptide from surface protein ClfB (SP_ClfB_) and a non-YSIRK signal peptide from a secreted serine protease SplE (SP_SplE_). Similar to the cross-wall IF results of SP_SpA_-SpA, SP_ClfB_-SpA localized at the cross-wall in SEJ1 WT but mislocalized in Δ*gdpP*. In contrast, SP_SplE_-SpA was distributed circumferentially in WT, and Δ*gdpP* did not affect its localization (Fig. 1A and B). The membrane localization of all three constructs (SP_SpA_-SpA, SP_ClfB_-SpA, and SP_SplE_-SpA) was not significantly altered in Δ*gdpP* (Fig. 1C and D). Taken together, these results confirmed that Δ*gdpP* dysregulated SpA localization independent of LtaS. Unlike LtaS that affects both cross-wall and septal membrane localization of SpA (56), deletion of *gdpP* diminishes cross-wall localization of SpA, and the phenotype is dependent on its YSIRK+ signal peptide.

### Δ*gdpP* cells exhibit aberrant Bocillin-FL foci that are predominantly found in non-dividing cells

**Fig 2:**
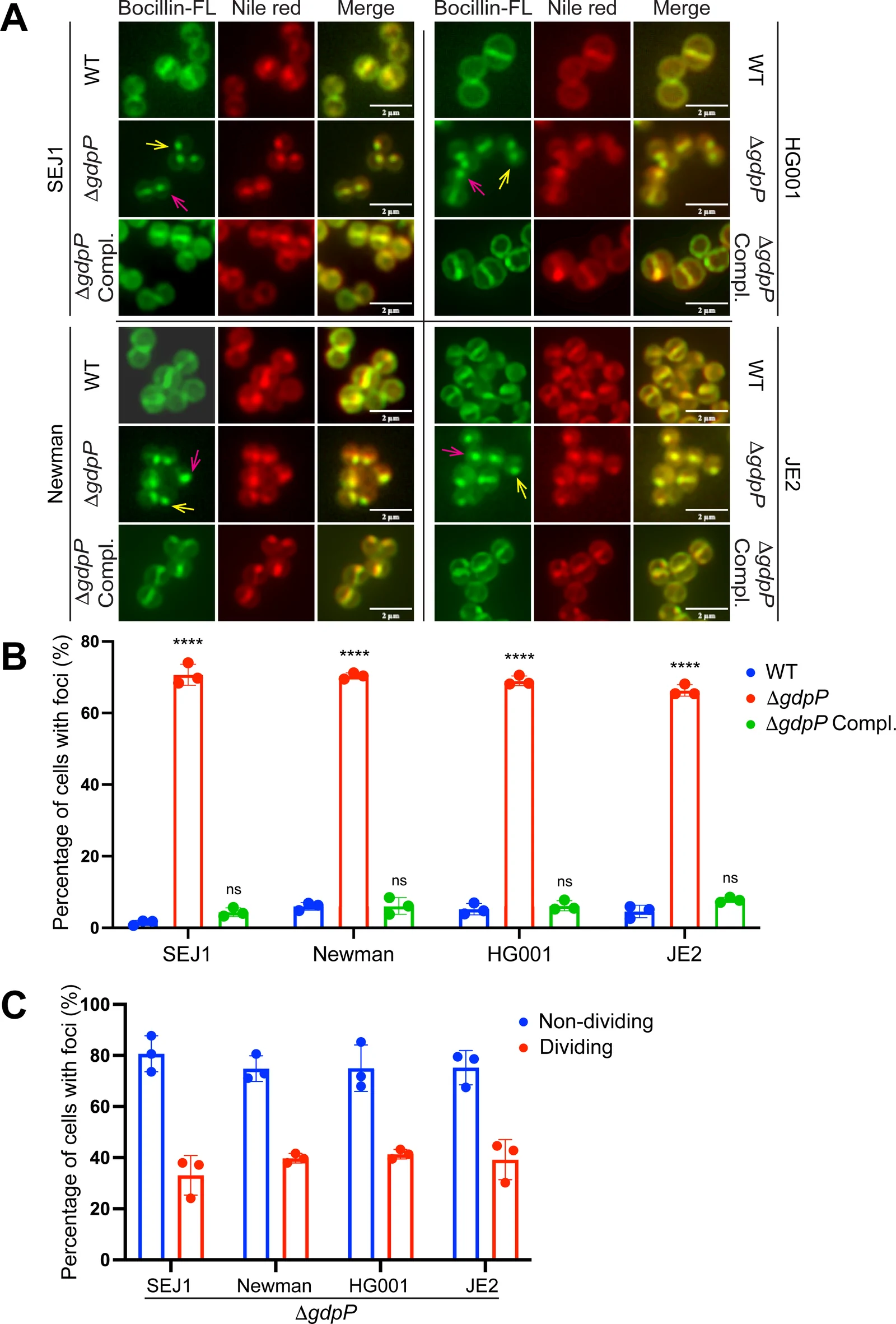
Aberrant Bocillin-FL foci formation in MSSA and MRSA Δ*gdpP*. (**A**) Fluorescence microscope images of Bocillin-FL staining of WT, Δ*gdpP*, and its complementation in different *S. aureus* strains: SEJ1, Newman, HG001, USA300 JE2. SEJ1, Newman, and HG001 are MSSA, and USA300 JE2 is MRSA. Nile red stains the cytoplasmic membrane. Scale bar, 2 µm. (**B**) Quantification of foci formation in cells from panel **A**. Unpaired *t*-test with Welch’s correction was performed for statistical analysis: ns, not significant; ****P < 0.0001. Representative images and quantification are from three independent experiments. (**C**) Quantification of foci formation in dividing and non-dividing cells in Δ*gdpP*.

The cross-wall trafficking of YSIRK+ proteins is coupled with cell cycle and cell wall homeostasis, which involves dynamic and balanced synthesis and degradation. Since Δ*gdpP* is resistant to β-lactam antibiotics that target PBPs, we prioritized our research to investigate PBP-mediated PG synthesis. We first performed Bocillin-FL staining to examine the localization of PBPs in the *gdpP* mutant. Bocillin-FL is a fluorescent penicillin that binds to the transpeptidase domain of all PBPs (68). As expected, Bocillin-FL signals were enriched at the septa of dividing cells (displayed as a septal line or septal ring) and distributed circumferentially along the cell membrane in non-dividing cells in *S. aureus* WT cells (Fig. 2A). Strikingly, the Δ*gdpP* cells exhibited distinct and strong Bocillin-FL foci formation, which appeared as one bright spot at the membrane. The aberrant Bocillin-FL foci formation was frequently found in non-dividing cells (Fig. 2A, yellow arrows) and less frequent in dividing cells. In most dividing cells, proper septum-localized Bocillin-FL signals could be observed. In some dividing cells, bright Bocillin-FL spots were observed at the edge or in the middle of the septal line (Fig. 2A, pink arrows). Interestingly, almost in all cases, each cell contained only one Bocillin-FL punctum, suggesting that one or more PBPs aggregated into one single microdomain in each cell. Quantification data revealed that 65–70% of Δ*gdpP* cells contain aberrant Bocillin-FL foci, whereas the incidence was very low in WT cells (2–5%) (Fig. 2B). Furthermore, 75–80% of non-dividing cells showed foci formation, whereas the rate was 35–40% in dividing cells (Fig. 2C). The results were consistent in all strains tested, including MSSA and MRSA, and can be complemented. Since the Δ*gdpP* cells grow slower than the WT, we examined the cells from different time points during growth (Fig. S3). The aberrant Bocillin-FL foci formation was observed at various time points (4, 6, 8, and 24 h), indicating that the foci formation was not due to slow growth. Collectively, the results demonstrate that PBPs are mislocalized as single foci in Δ*gdpP* cells; the foci are predominantly found in non-dividing cells and are independent of growth phase.

### Δ*gdpP* cells are deficient in cell division initiation

**Fig 3:**
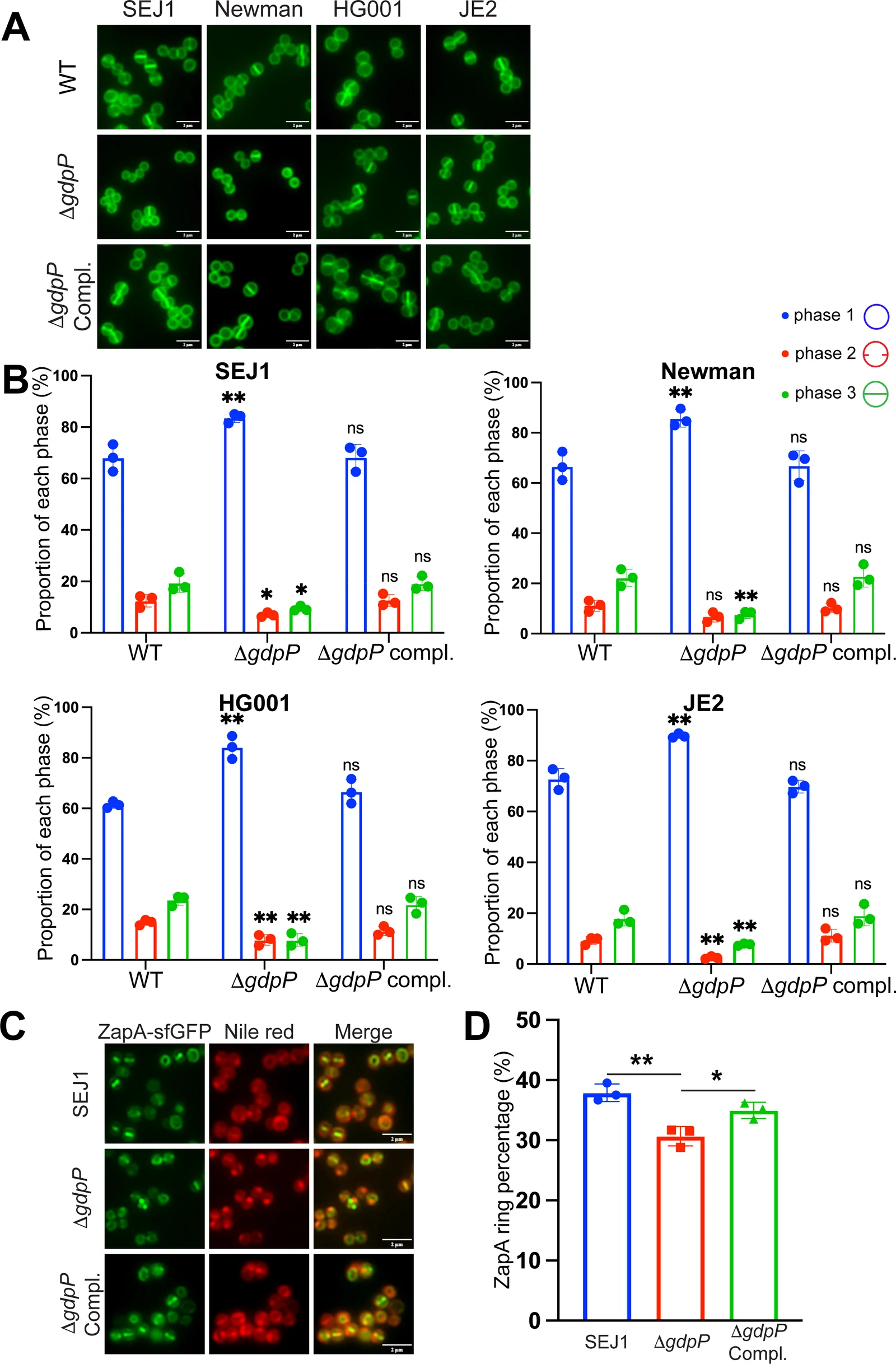
Δ*gdpP* is deficient in cell division initiation with decreased ZapA septal ring formation. (**A**) Van-FL labeling of WT, Δ*gdpP*, and *gdpP* complementation in *S. aureus* SEJ1, Newman, HG001, and JE2. Scale bar, 2 µm. (**B**) Quantification of cells at different cell cycle stages: with no septum (P1), a partial septum (P2), or a complete septum (P3). Asterisks on top of each sample indicate the *P* value when comparing the sample with the corresponding phase of WT cells. Unpaired *t*-test with Welch’s correction was performed for statistical analysis. (**C**) The localization of ZapA-GFPmut2 in SEJ1 WT, SEJ1Δ*gdpP*, and *gdpP* complementation. Nile red stains cytoplasmic membrane. Scale bar, 2 µm. (**D**) Quantification of ZapA septal ring formation. Unpaired *t*-test with Welch’s correction was performed for statistical analysis: ns, not significant; *P < 0.05; **P < 0.005. Representative images and quantification are from three independent experiments.

From the above experiments, we noticed that most of the Δ*gdpP* cells were not dividing. To further investigate this, we performed cell cycle analysis. The cell cycle of staphylococcal cells has been defined as three phases (63, 69): phase 1 cells exhibit no septa, having completed their previous cell division and not yet started the next cycle; phase 2 cells exhibit incomplete septa, having just initiated cell division; phase 3 cells have completed septa and exhibit an elongated shape. Compared to the WT, Δ*gdpP* showed a 15–20% increase in the phase 1 cell population, and a 5% and 10–15% decrease in the phase 2 and phase 3 cell populations. The alteration was observed in all strain backgrounds and was statistically significant (Fig. 3A and B). The accumulation of phase 1 non-dividing cells strongly indicates a defect in cell division initiation. To further analyze cell division initiation, we examined ZapA-GFPmut2 localization (Fig. 3C and D). ZapA is an early cell division protein that promotes FtsZ polymerization and divisome formation (55, 70, 71). The localization of ZapA indicates the sites of divisome formation at the early stage of cell division. Microscopy and quantification analysis revealed that the frequency of ZapA septal localization (ZapA ring formation) was decreased by 7% in Δ*gdpP* compared to WT*,* indicating a defect in cell division initiation. Note that we only observed a reduction in ZapA septal ring formation but did not observe aberrant ZapA localization in Δ*gdpP*. The decrease in ZapA septal ring formation (7%) is not as dramatic as the decrease in dividing cells (15–20%), suggesting that additional mechanisms may contribute to the cell division defect of Δ*gdpP*. In conclusion, the Δ*gdpP* cells are deficient in cell division initiation and cell cycle progression, leading to the accumulation of non-dividing cells.

### The aberrant Bocillin-FL foci formation is due to c-di-AMP accumulation

**Fig 4:**
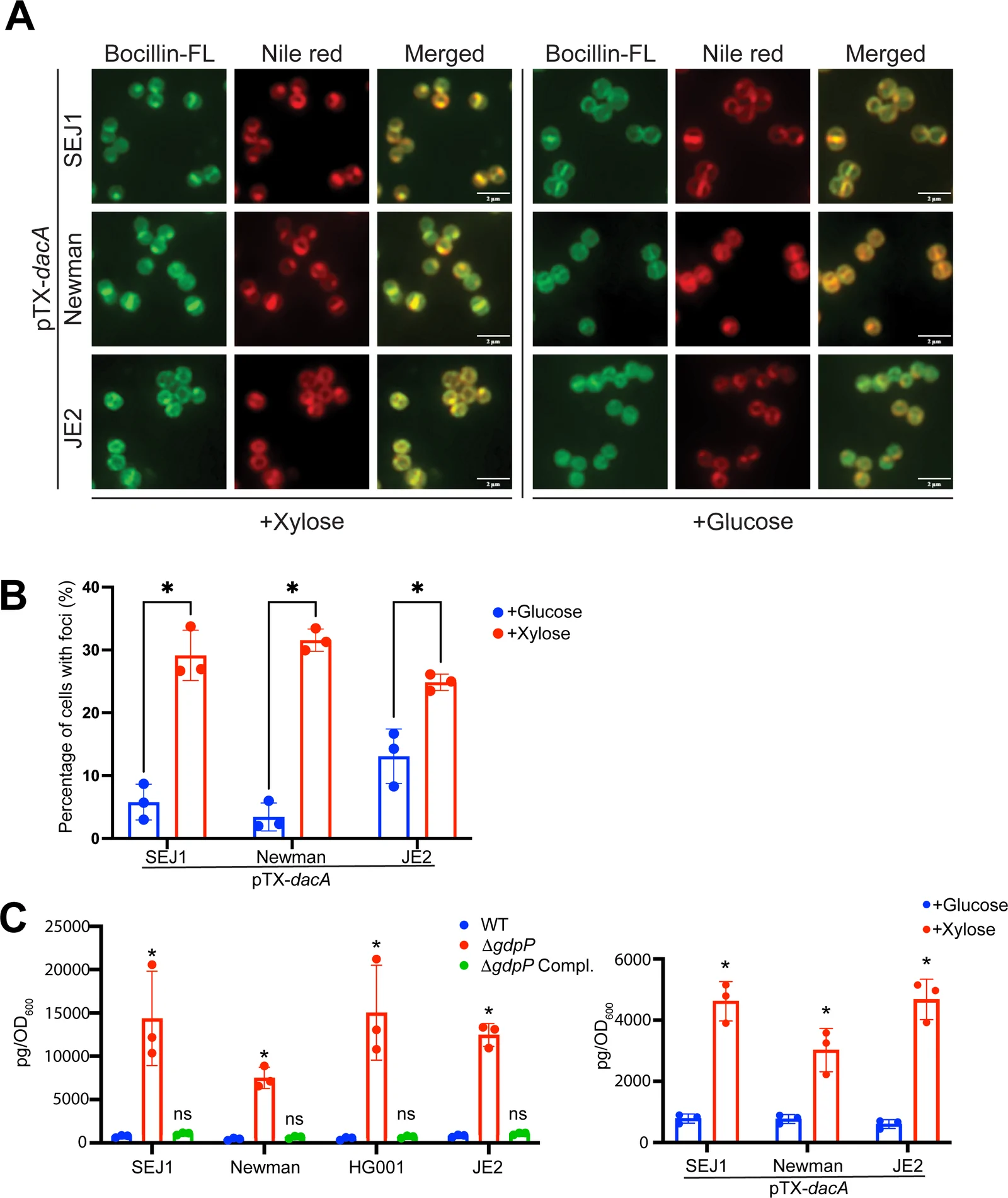
Overexpression of *dacA* leads to aberrant Bocillin-FL foci formation. (**A**) *dacA* overexpressed (+xylose) and repressed (+glucose) cells were stained with Bocillin-FL. Nile red stains cytoplasmic membrane. Scale bar, 2 µm. (**B**) Quantification of foci formation from panel **A**. Unpaired *t*-test with Welch’s correction was performed for statistical analysis: *P < 0.05. Representative images and quantification are from three independent experiments. (**C**) C-di-AMP levels in WT, Δ*gdpP*, *gdpP* complementation, and *dacA* OE strains. Unpaired *t*-test with Welch’s correction was performed for statistical analysis: ns, not significant; *P < 0.05. The results are from three independent experiments.

Next, we investigated whether the Bocillin-FL foci formation was due to mutation of *gdpP* or accumulation of c-di-AMP. To test this, we overexpressed the c-di-AMP synthase gene *dacA* in SEJ1, Newman, and JE2 via a xylose-inducible promoter from a multicopy plasmid. As a control, glucose was added to repress the expression of *dacA*. Overexpression of *dacA* resulted in smaller cell size like Δ*gdpP* and a significant increase in Bocillin-FL foci formation in all strains tested (Fig. 4A). Like Δ*gdpP,* the foci were predominantly found in non-dividing cells. However, the rate of foci formation upon *dacA* overexpression (25–32%) was lower than that of Δ*gdpP* (65–70%) (Fig. 4B). To examine whether foci formation correlates with the level of c-di-AMP accumulation, we measured the intracellular c-di-AMP concentration in WT, Δ*gdpP*, *gdpP* complementation, and *dacA* overexpression strains. As shown in Fig. 4C, Δ*gdpP* accumulated a significant amount of c-di-AMP compared to WT (15- to 30-fold increase). Overexpression of *dacA* also led to c-di-AMP accumulation but to a lesser degree (4- to 8-fold increase), likely because GdpP is still functional in these strains to degrade c-di-AMP. In conclusion, the level of Bocillin-FL foci formation correlates with the level of c-di-AMP accumulation; it is not the *gdpP* mutation *per se*, but the accumulation of c-di-AMP that leads to Bocillin-FL foci formation.

### All four PBPs are mislocalized as single foci at the cell periphery of Δ*gdpP* cells

**Fig 5:**
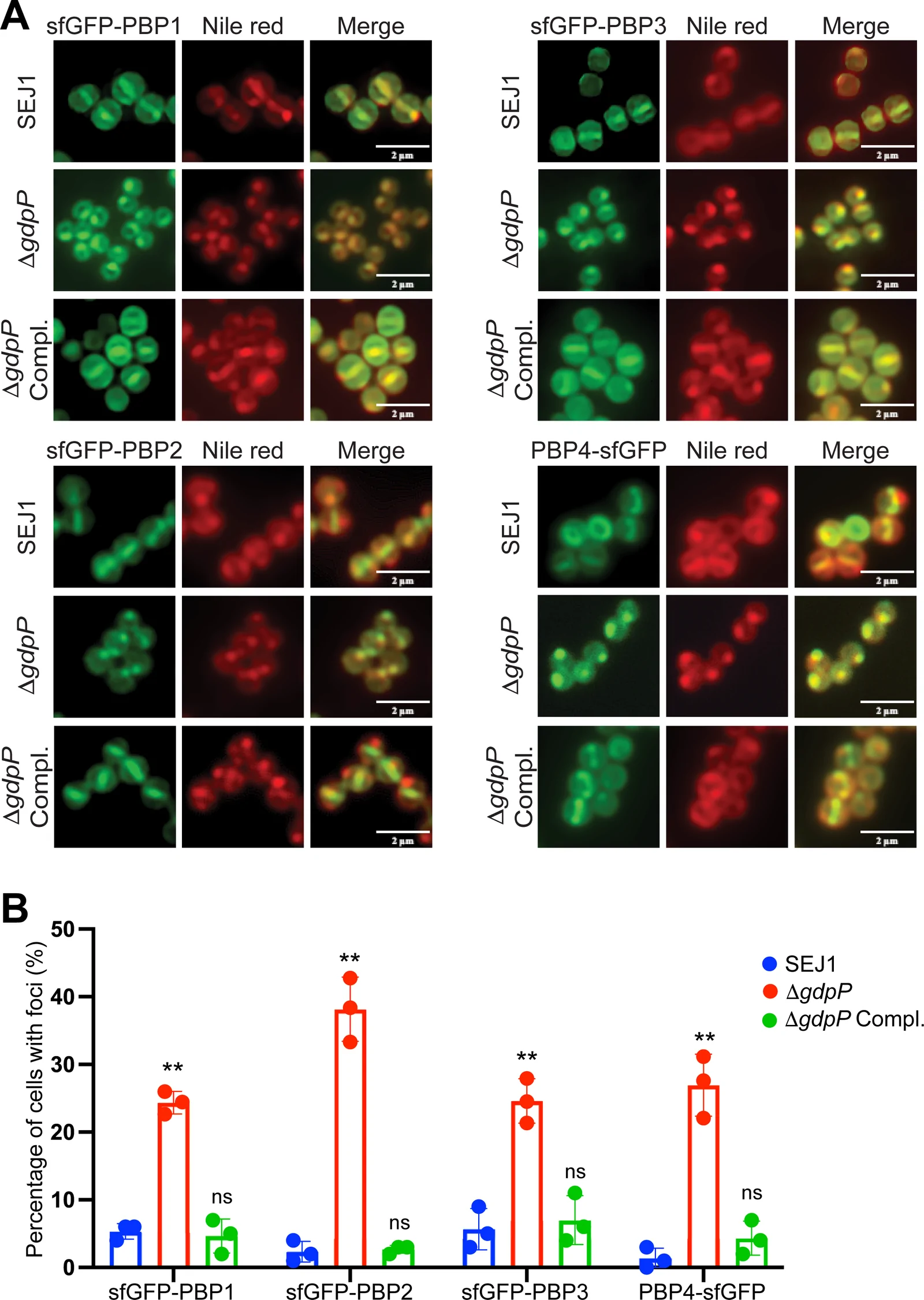
All four native PBPs (PBP1-4) form aberrant foci in Δ*gdpP* cells. (**A**) Fluorescence microscope images of sfGFP-PBP1, sfGFP-PBP2, sfGFP-PBP3, and PBP4-sfGFP in SEJ1 WT, Δ*gdpP*, and *gdpP* complementation strains. Nile red stains cytoplasmic membrane. Scale bar, 2 µm. (**B**). Quantification of PBP foci formation from the images in panel **A**. Unpaired *t*-test with Welch’s correction was performed for statistical analysis: ns, not significant; **P < 0.005. Representative images and quantification are from three independent experiments.

The genome of *S. aureus* encodes four PBPs, namely PBP1, PBP2, PBP3, and PBP4 (72–74). To further investigate which PBPs were mislocalized in Δ*gdpP*, PBP1–4 were individually tagged with a superfolder green fluorescent protein (sfGFP) and expressed from an ATc-inducible promoter from the chromosome. Consistent with previous studies (72–74), all four PBPs localized at the septa of dividing cells and distributed over the cell periphery in non-dividing cells in WT. Intriguingly, all four PBPs formed distinct foci in Δ*gdpP* cells (Fig. 5A). Similar to the results of Bocillin-FL staining, the foci of PBPs were deposited as single puncta at one site of the membrane in each cell, which were predominantly found in non-dividing cells. Nearly 40% of cells displayed sfGFP-PBP2 foci, which is the highest among all four PBPs. SfGFP-PBP1, sfGFP-PBP3, and PBP4-sfGFP showed a lower percentage (about 25%) of foci formation in Δ*gdpP*, but all were significantly higher than that of WT (2–5%) (Fig. 5B). The percentage of PBP foci formation is lower than that of Bocillin-FL foci, which could be due to the higher stability and sensitivity of Bocillin-FL staining compared to GFP-tagging. Complementation of *gdpP* restored the localization of PBPs comparable to the WT. Collectively, the results suggest that all four native PBPs aggregate into aberrant single foci in Δ*gdpP* cells.

### The PBPs aggregated in the peripheral foci are largely inactive, whereas septal localized PBPs are active to incorporate FDAAs

**Fig 6:**
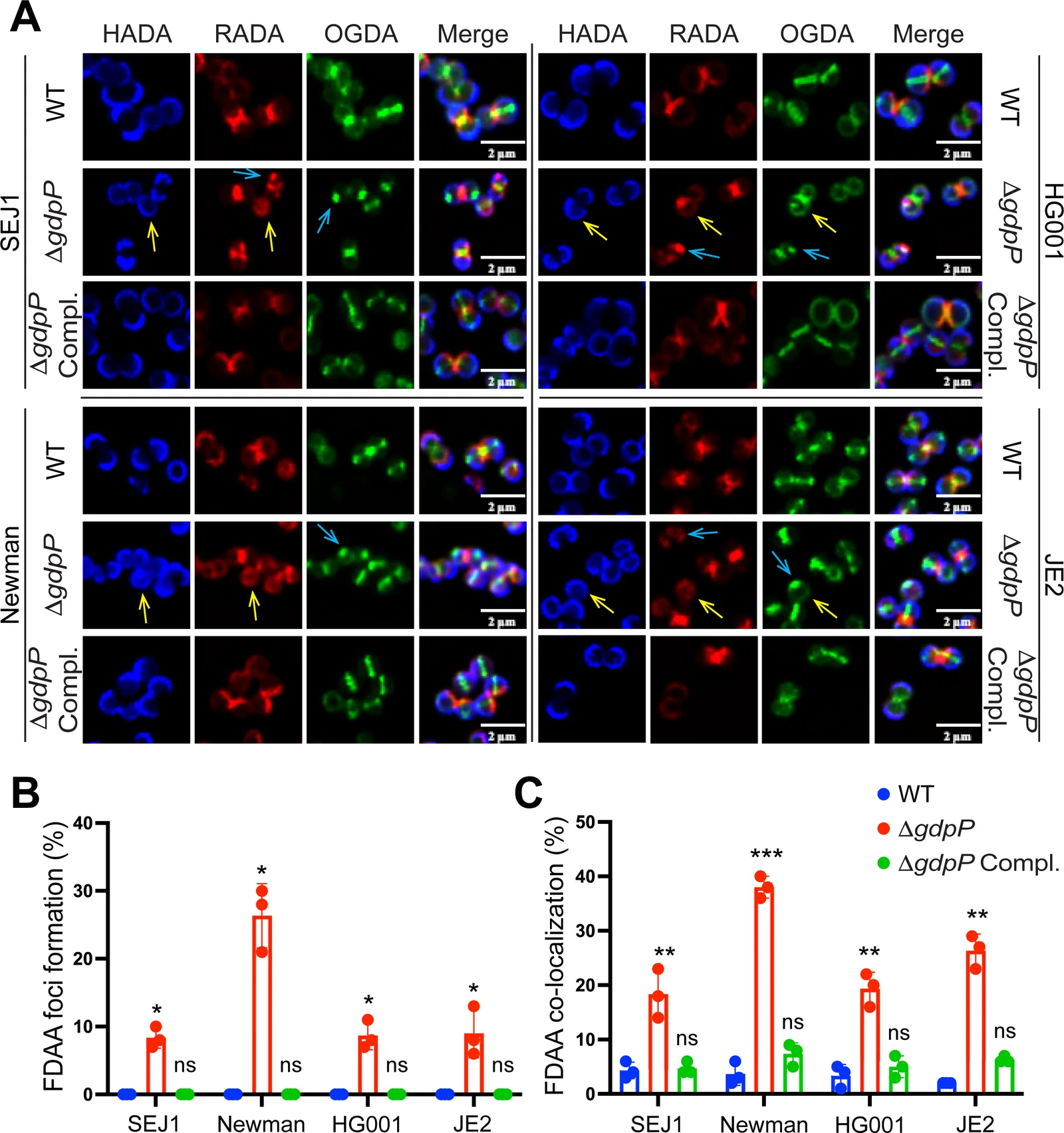
PBP-mediated FDAA incorporation during cell cycle. (**A**) *S. aureus* WT, Δ*gdpP*, and *gdpP* complementation strains were sequentially incubated with three FDAAs: HADA (blue), RADA (red), and OGDA (green), and imaged by fluorescence microscope. Scale bar, 2 µm. Blue arrows indicate FDAA mislocalization as foci; yellow arrows indicate co-localization of FDAAs. (**B**) Quantification of FDAA foci formation. (**C**) Quantification of FDAA co-localization. Unpaired *t*-test with Welch’s correction was performed for statistical analysis: ns, not significant; *P < 0.05; **P < 0.005; ***P < 0.0005. Representative images and quantification are from three independent experiments. Additional larger images are shown in Fig. S4.

Next, we tested the activity of PBPs by fluorescent d-amino acid (FDAA) incorporation experiment. The FDAAs are actively incorporated into the cell wall via the transpeptidase activity of PBPs, and the method has been widely used to label active cell wall synthesis sites subcellularly (63, 75–77). In our experiment, we sequentially labeled the *S. aureus* cells with three fluorescent d-amino acids (FDAAs) along the cell cycle (63). In WT *S. aureus* cells, HADA (blue) stains the old peripheral wall of the two daughter cells (half-moon shape), RADA (red) stains the cross-wall between the two daughter cells (“Y” or “V” shape), and OGDA (green) stains the newest cross-wall synthesis within the two daughter cells (straight lines) (Fig. 6; Fig. S4). Compared to WT, most Δ*gdpP* cells showed normal FDAA septal incorporation, suggesting that septal localized PBPs are functional. Some Δ*gdpP* cells showed abnormal phenotypes: (i) aberrant FDAA foci (10–25% in Δ*gdpP* cells) (Fig. 6; Fig. S4, blue arrows) or (ii) delayed FDAA incorporation shown as co-localization of at least two FDAAs (20–40% in Δ*gdpP* cells) (Fig. 6; Fig. S4, yellow arrows). We reasoned that the aberrant foci of FDAAs were incorporated by aggregated PBP, and the delayed FDAA incorporation was very likely due to cell cycle retardation. However, the rate of FDAA foci (10–25%) is significantly lower than that of PBP/Bocillin-FL foci (65–70%) (Fig. 2). If the PBPs in the foci were fully active to incorporate FDAA, the rate of FDAA foci would be close to that of PBP foci. Yet this is not the case, suggesting that most PBPs in the foci are inactive and unable to incorporate FDAA locally.

### The overall PG cross-linking is not significantly altered in Δ*gdpP* cells

**Fig 7:**
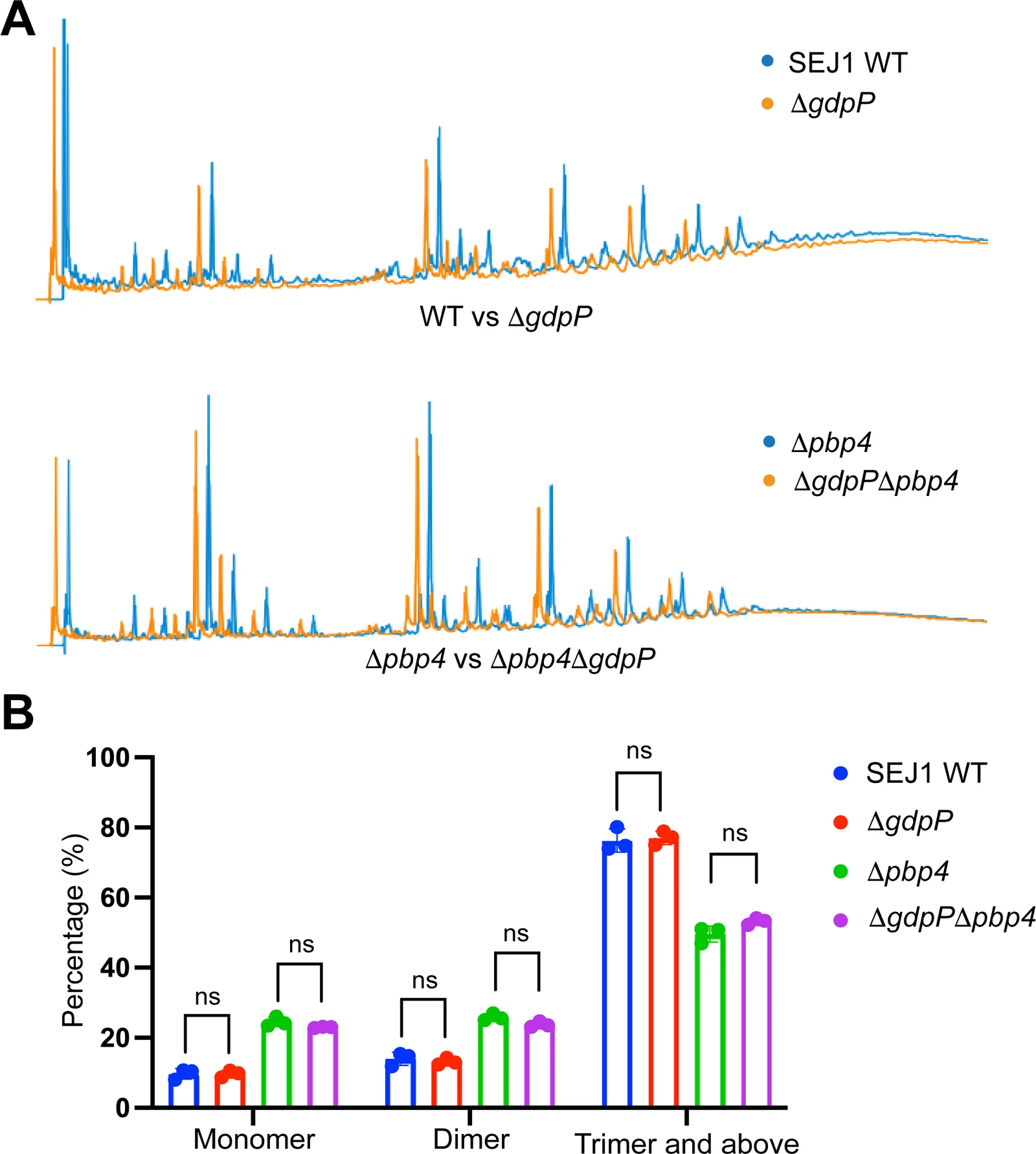
Cell wall PG cross-linking is not altered in Δ*gdpP*. (**A**) HPLC analysis of PG cross-linking of SEJ1 WT, Δ*gdpP*, Δ*pbp4*, and Δ*gdpP*Δ*pbp4*. (**B**) Quantification of the area under the curve of monomers, dimers, and trimers, and above from panel **A**. Unpaired *t*-test with Welch’s correction was performed for statistical analysis: ns, not significant. The results are from three independent experiments.

Next, we examined the overall PG cross-linking of the *gdpP* mutants. PG analysis from previous studies showed inconsistent results: Δ*gdpP* was reported to have increased PG cross-linking in an earlier study, while a recent study showed no difference (13, 23). Since PBP4 is the primary contributor for highly cross-linked PG in *S. aureus* (78, 79), we included Δ*pbp4* and generated a Δ*gdpP*Δ*pbp4* double mutant. The cell wall peptidoglycan from SEJ1 WT, Δ*gdpP*, Δ*pbp4*, and Δ*gdpP*Δ*pbp4* was purified, digested with mutanolysin (N-acetylmuramidase), and analyzed by high-performance liquid chromatography (HPLC). Δ*gdpP* showed a slight increase in PG cross-linking, but quantification and statistical analysis revealed no significant differences (Fig. 7). As expected, Δ*pbp4* exhibited a significant decrease in PG cross-linking. There was a small increase in PG cross-linking in Δ*gdpP*Δ*pbp4* compared to Δ*pbp4*, but the increase was not statistically significant. Overall, the results suggested that Δ*gdpP* did not significantly alter the overall PG cross-linking. In line with the results from PBP localization and FDAA incorporation, we concluded that the aggregated PBPs in the foci at the cell periphery are mostly inactive, whereas the septal localized PBPs in dividing cells are active, and their activity is sufficient to maintain overall PG cross-linking in a cell population.

### The PBP foci correlate with resistance to β-lactams that target PBPs, but not to vancomycin that targets PG cross-linking

Mutations in the *gdpP* gene have been reported to cause β-lactam resistance and tolerance in the literature (12–25). To examine antibiotic resistance, we measured the MIC of three β-lactams (penicillin G, oxacillin, and nafcillin) and other cell envelope-targeting antibiotics (nisin, vancomycin, and daptomycin). To rule out potential interference due to the slow growth of Δ*gdpP*, we tested MIC with extended incubation times (24 and 48 h). The MIC values were the same after 24 and 48 h of incubation. As shown in Table 1, the *gdpP* mutants were resistant to all three β-lactams tested: the MIC values consistently increased by 4- to 8-fold in both MSSA and MRSA Δ*gdpP* strains. The results confirmed that *gdpP* mutation triggered additional resistance independent of *mecA* in MRSA. In contrast to β-lactams, the MIC values for other cell envelope-targeting antibiotics did not change significantly in Δ*gdpP*. To examine antibiotic tolerance, a tolerance disk test (TDtest) was performed (Fig. S5). TDtest is a modified disk-diffusion assay widely used to measure antibiotic tolerance (23, 80). Specifically, following incubation with an antibiotic disk, the disk is replaced by a disk with glucose to allow tolerant colonies to grow (23, 80). The level of tolerance is quantified by counting the colonies grown in the inner-half inhibition zone close to the glucose disk. As shown in Fig. S5, SEJ1 WT and JE2 WT did not show tolerance toward β-lactams or vancomycin; SEJ1Δ*gdpP* showed various numbers of tolerant colonies toward different β-lactams; however, JE2Δ*gdpP* did not show tolerance toward any β-lactams tested. Moreover, none of the strains showed tolerance toward vancomycin. These results indicate that β-lactam resistance is a conserved phenotype, whereas tolerance is strain-dependent. Since the aberrant foci formation of PBPs was observed in both MSSA and MRSA, it correlates with resistance, not tolerance.

**TABLE 1:** MIC of WT, Δ*gdpP*, and complementation strains toward β-lactams and other cell envelope-targeting antibiotics^*a*^

Strain	MIC (µg/mL)					
	PCN	OXA	NAF	NIS	VAN	DAP
SEJ1 WT	0.0625	0.5	8	256	2	0.5
SEJ1Δ*gdpP*	0.25	2	64	128	2	0.5
SEJ1Δ*gdpP* pKK30-*gdpP*	0.125	1	16	256	2	0.5
JE2 WT	4	32	256	256	2	1
JE2Δ*gdpP*	16	256	2048	256	2	1
JE2Δ*gdpP* pKK30-*gdpP*	8	64	1024	256	2	1

^*a*^ DAP, daptomycin; NAF, nafcillin; NIS, nisin; OXA, oxacillin; PCN, penicillin G; VAN, vancomycin..

**Fig 8:**
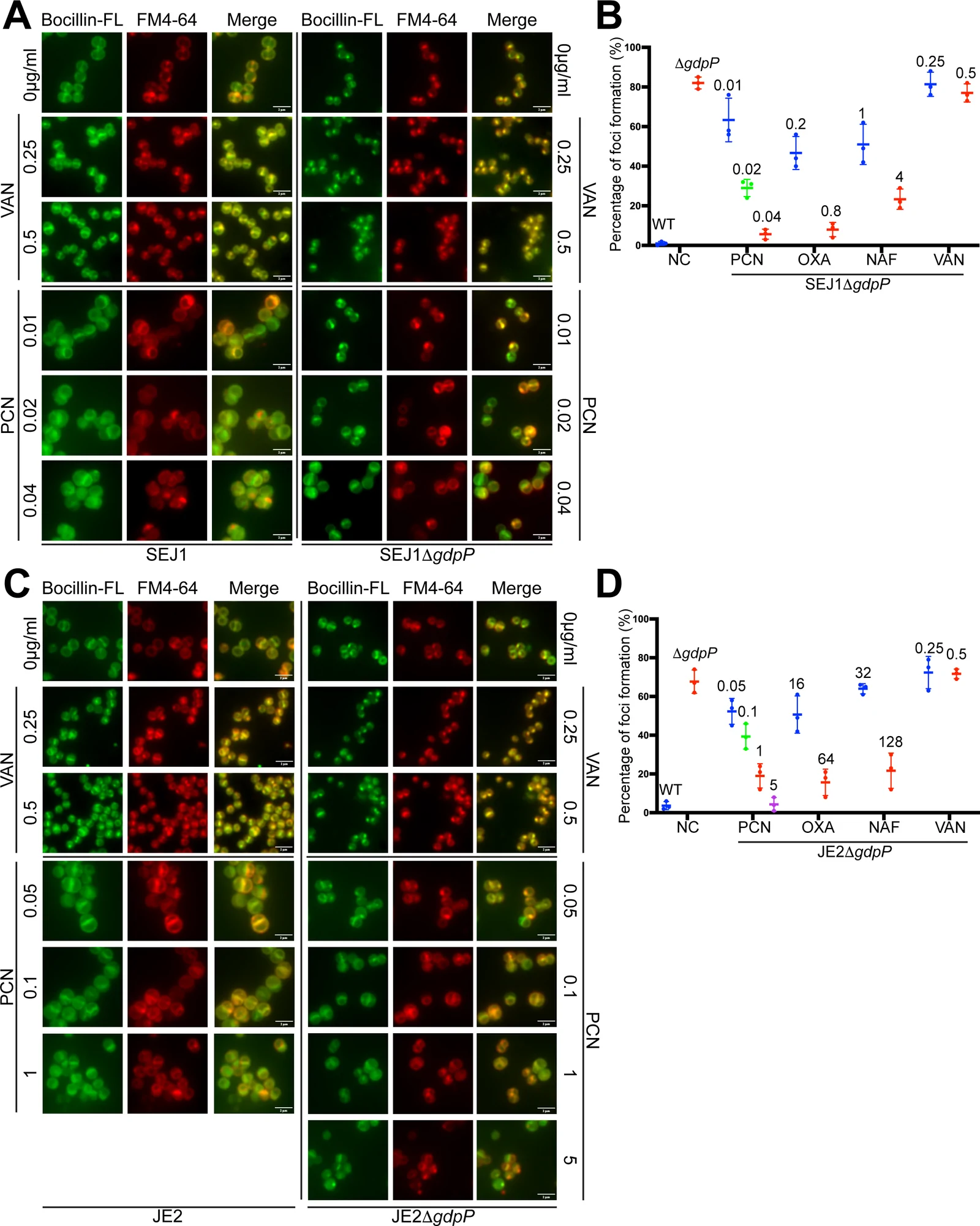
The aberrant foci formation of PBPs correlates with β-lactam resistance. (**A and C**) Bocillin-FL staining of SEJ1 WT (MSSA), SEJ1Δ*gdpP*, JE2 WT (MRSA), and JE2Δ*gdpP* after treatment with different concentrations of penicillin G (PCN) and vancomycin (VAN). FM4-64 stains cytoplasmic membrane. Scale bar, 2 µm. The concentrations of antibiotics (µg/mL) are indicated. (**B and D**) quantification of Bocillin-FL foci formation upon antibiotic treatment: no antibiotics (NC), penicillin G (PCN), vancomycin (VAN), oxacillin (OXA), and nafcillin (NAF). Images of OXA and NAF treatment are shown in Fig. S6.

To further investigate the connection between PBP foci formation and β-lactam resistance, we treated MSSA SEJ1 and MRSA JE2 WT and Δ*gdpP* with three β-lactams (penicillin G, oxacillin, and nafcillin) and vancomycin, and then stained the cells with Bocillin-FL. We included vancomycin because both β-lactams and vancomycin inhibit PG cross-linking, albeit with different mechanisms: β-lactams target PBP’s transpeptidase activity, whereas vancomycin binds to the d-alanyl-d-alanine terminus of the muropeptides. Furthermore, Δ*gdpP* was resistant to β-lactams but not to vancomycin (Table 1). Intriguingly, the foci formation of PBPs correlated with various concentrations of β-lactams but was insensitive to vancomycin (Fig. 8; Fig. S6). Figure 8 shows the results of penicillin as a representative of β-lactams. Treatment with lower concentrations of penicillin (0.01 µg/mL for SEJ1 and 0.05 µg/mL for JE2) in the WT cells readily triggered enlarged cell size and aberrant division septa formation. These morphological changes indicated the killing activity of penicillin in weakening the cell wall and inducing cell lysis. In comparison, low concentrations of penicillin did not alter the morphology or the Bocillin-FL foci formation of Δ*gdpP* cells. Increasing penicillin concentrations gradually triggered morphological changes and killing of the Δ*gdpP* cells. Notably, the Bocillin-FL foci formation gradually decreased with increasing concentrations of penicillin. At high concentrations of penicillin (0.04 µg/mL for SEJ1Δ*gdpP* and 5 µg/mL for JE2Δ*gdpP*), the Δ*gdpP* cells were significantly enlarged with irregular septa, and the Bocillin-FL foci completely disappeared, indicating that high concentrations of penicillin disrupted the Bocillin-FL foci and triggered killing. Thus, the presence of Bocillin-FL foci formation correlated with resistance to penicillin-mediated killing. The same results were observed with oxacillin and nafcillin treatment and in both MSSA and MRSA strains (Fig. 8B and D; Fig. S6). In contrast, vancomycin treatment did not affect the PBP foci formation at various concentrations tested, while the bacterial growth was severely inhibited (Fig. 8). Collectively, PBP foci formation correlates with resistance to β-lactams that directly bind to PBPs, but not to vancomycin that binds to Ala-d-Ala and PG cross-linking.

### Δ*gdpP* cells exhibit increased binding to Bocillin-FL

**Fig 9:**
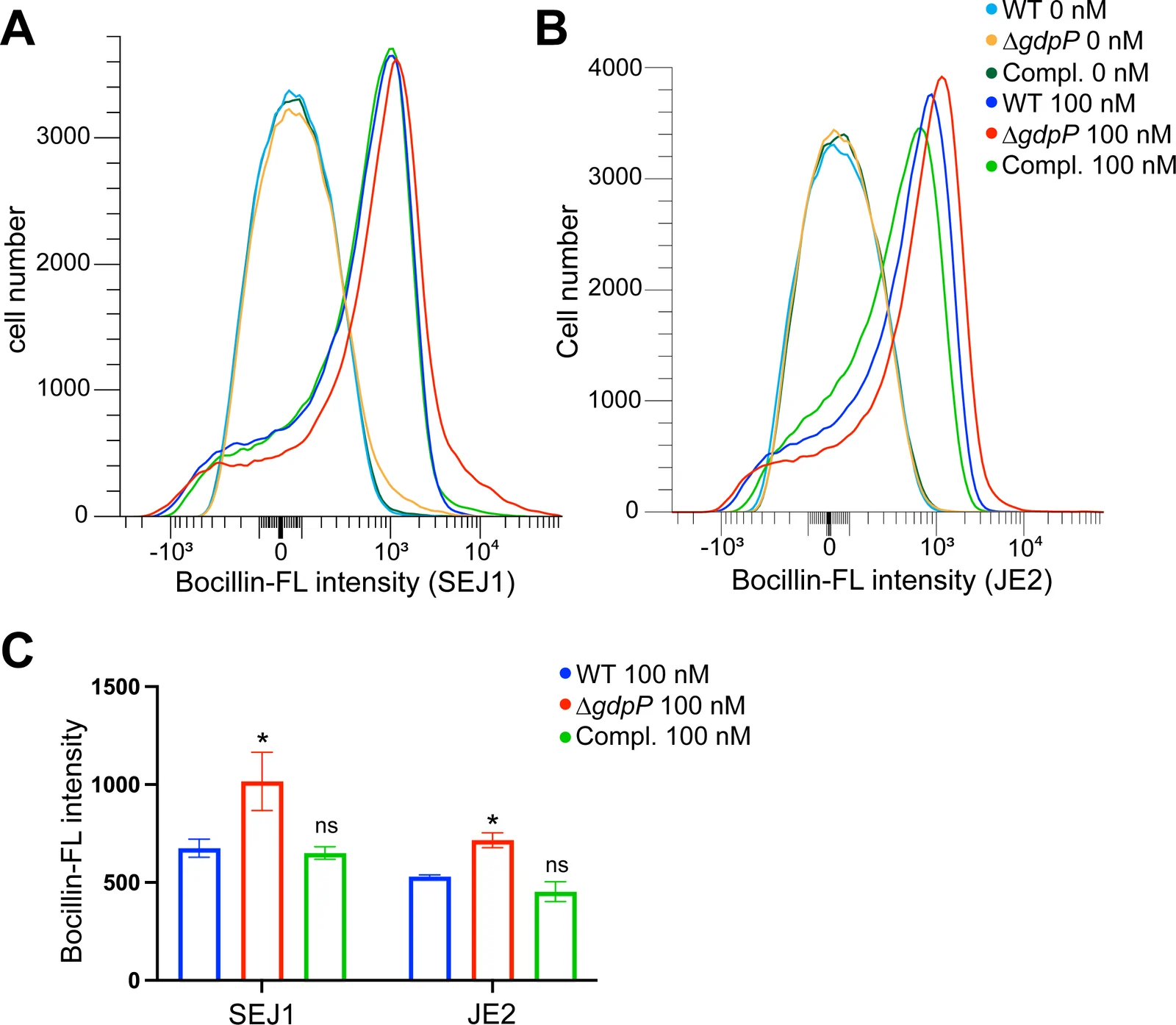
Δ*gdpP* cells exhibit increased binding to Bocillin-FL. *S. aureus* WT, Δ*gdpP*, and *gdpP* complementation cells were incubated with 0 and 100 nM Bocillin-FL, washed, and analyzed by flow cytometry. (**A**) Bocillin-FL fluorescence signals in the populations of SEJ1 WT, SEJ1Δ*gdpP*, and its complementation strains. (**B**) Bocillin-FL fluorescence signals in the populations of JE2 WT, JE2Δ*gdpP*, and its complementation strains. (**C**) Quantification of average Bocillin-FL intensity in SEJ1 and JE2 WT, Δ*gdpP*, and *gdpP* complementation cells. Unpaired *t*-test with Welch’s correction was performed for statistical analysis: ns, not significant; *P < 0.05. The results are from three independent experiments.

Finally, we tested the direct binding of Δ*gdpP* cells to β-lactams. *S. aureus* WT, Δ*gdpP*, and its complementation strains were incubated with Bocillin-FL and subjected to flow cytometry analysis. We were concerned that the Δ*gdpP* cells were smaller, which may introduce errors in quantifying Bocillin-FL fluorescence. Flow cytometry allows us to minimize the potential artifacts by selecting the equivalent single-cell populations via cell size scattering and measuring fluorescence distribution per cell number in a population. Figure 9A and B show Bocillin-FL fluorescence distribution in the populations of WT, Δ*gdpP*, and its complementation in SEJ1 and JE2 backgrounds. Interestingly, both SEJ1Δ*gdpP* and JE2Δ*gdpP* exhibited increased binding to Bocillin-FL compared to the corresponding WT and complementation strains. Quantification results showed that the average fluorescence intensity of the Δ*gdpP* cells was higher than that of WT and complementation strains (50% increase in SEJ1Δ*gdpP* and 35% increase in JE2Δ*gdpP*) (Fig. 9C). These results suggested that Δ*gdpP* cells bind more β-lactams compared to WT.

## DISCUSSION

**Fig 10:**
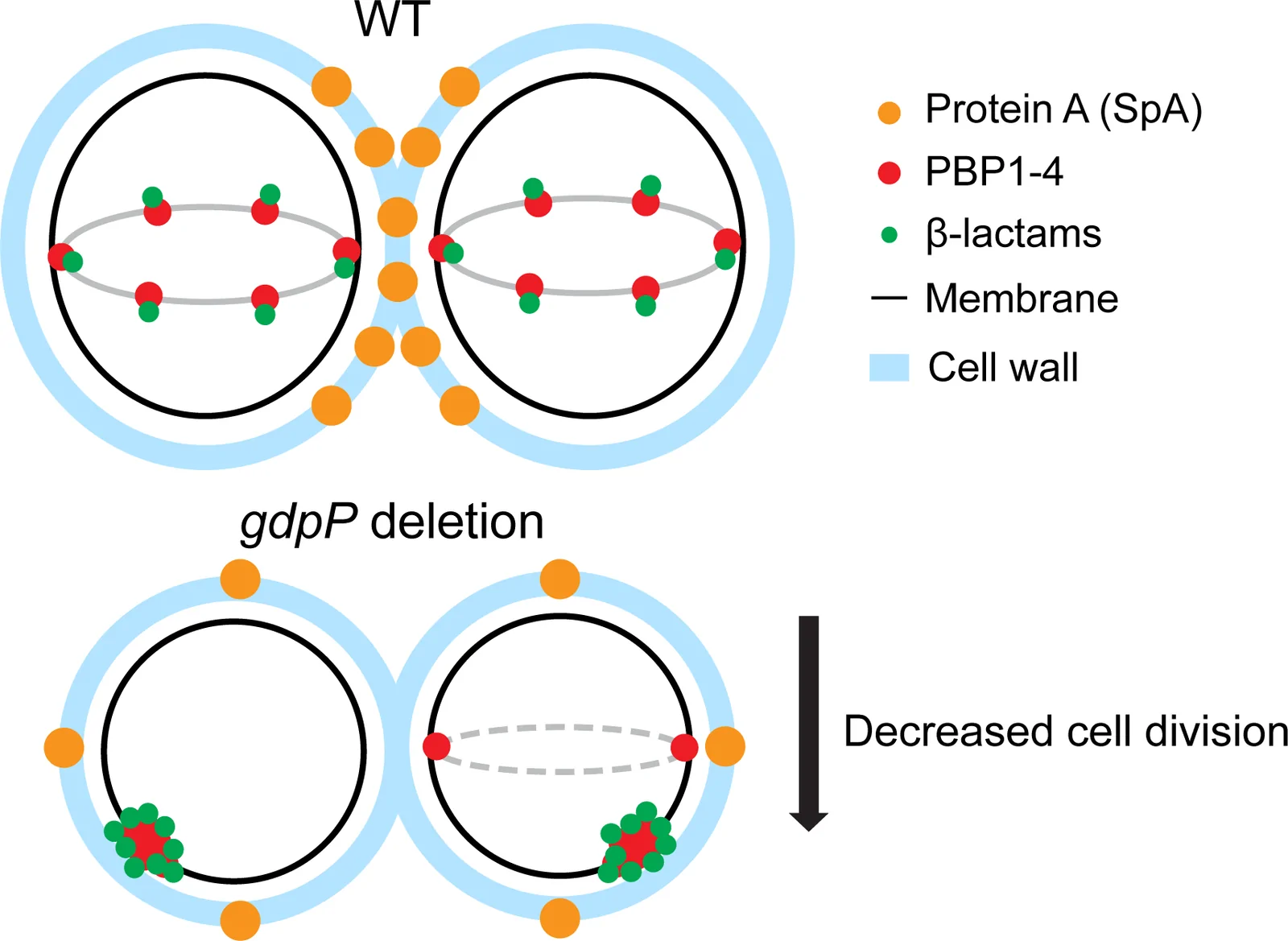
Model of how Δ*gdpP* affects β-lactam resistance and surface virulence protein assembly. In WT staphylococcal cells, PBP1–4 are localized at the septum of dividing cells to synthesize the cross-wall where YSIRK+ surface protein SpA is enriched. In Δ*gdpP* cells, cell division is retarded, and PBPs accumulate as single foci at the cell periphery of non-dividing cells. The peripheral PBP foci mis-target β-lactams, and the cell division defect diminishes cross-wall assembly of SpA.

In summary, we showed here that *S. aureus* Δ*gdpP* diminished the cross-wall deposition of surface protein SpA in a YSIRK+ signal peptide-dependent manner. The *gdpP* mutant is deficient in cell division, leading to accumulation of non-dividing cells. Strikingly, all four PBPs mislocalize as single foci in Δ*gdpP* cells, mostly found in non-dividing cells. The aggregated PBPs in these foci are largely inactive as they are unable to incorporate FDAAs. The *gdpP* mutant did not show significant change in PG cross-linking and did not show resistance toward PG cross-linking targeting antibiotics such as vancomycin. The *gdpP* mutants were resistant to β-lactams, and increased concentrations of β-lactams are required to disrupt peripheral PBP foci to trigger killing. We propose that the aggregation of PBPs at the cell periphery and the cell division defect of Δ*gdpP* contribute to β-lactam resistance and dysregulate SpA cross-wall trafficking (Fig. 10): the aberrant PBP foci mis-target β-lactams to the cell periphery, and the accumulation of non-dividing cells further enhances the “off target” effect of β-lactams. The mislocalization of SpA in Δ*gdpP* cells is likely due to delayed cell cycle that accumulates peripheral SpA. While the *gdpP* mutant accumulates non-dividing cells and PBPs primarily aggregate in non-dividing cells, most of the dividing cells have proper septal localized PBPs that actively synthesize the cross-wall and produce daughter cells, maintaining the overall PG cross-linking and viability in a cell population.

Cumulative studies have reported a correlation between the level of c-di-AMP with β-lactam susceptibility. In most cases, low c-di-AMP leads to hypersensitivity, and high c-di-AMP leads to decreased susceptibility. However, a recent study from *Listeria monocytogenes* reported that accumulation of c-di-AMP in the *pdeA pgpH* mutant increased susceptibility to β-lactams (cefuroxime) (52). Our results, that high levels of c-di-AMP render resistance, are consistent with previous studies in *S. aureus* (12–25). Furthermore, both resistance and tolerance have been reported with *gdpP* mutations (12–25). We observed that resistance was a conserved phenotype across MSSA and MRSA strains and toward different β-lactams, while tolerance was strain-specific. Antibiotic resistance is defined as the ability of bacteria to replicate in the presence of an antibiotic, which is measured by having a higher MIC value, whereas tolerance is defined as having the same MIC but an elongated time of killing (81, 82). Resistance can be caused by diverse mechanisms, but all lead to reduced antibiotic binding to the target (off target), while tolerance can be attributed to slow growth or extended lag phase (81, 82). We showed here that the MIC of Δ*gdpP* consistently increased by 4- to 8-fold toward various β-lactams, which remained the same after elongated incubation. Moreover, PBP foci formation was independent of slow growth. Our results, that PBPs aggregate and mislocalize β-lactams at the cell periphery, support an off-target resistance mechanism. On the other hand, tolerance was observed in the MSSA Δ*gdpP* strain, suggesting that there are mechanisms responsible for tolerance.

The mechanisms of how c-di-AMP signaling regulates susceptibility to β-lactams are largely unclear. As mentioned in the introduction, two models have been proposed: (i) c-di-AMP regulates internal turgor pressure, which indirectly affects cell integrity (13, 32, 51); and (ii) change in PG structure, including a thinner PG layer revealed by TEM (22), reduced PG content from PG purification (52), increased PG cross-linking (13), or change in PG precursors (52, 53). We think that decreased internal turgor pressure cannot explain all the phenotypes in Δ*gdpP* as the mutant is only resistant to β-lactams, not to vancomycin and other cell wall-targeting antibiotics. We did not observe significant changes in PG mass or PG overall cross-linking during our PG analysis. We present new findings here that all four PBPs are mislocalized as single foci in Δ*gdpP* cells, which correlates with β-lactam resistance. However, we have not been able to obtain causal evidence linking PBP foci to β-lactam resistance. Future work will be to disrupt PBP foci or introduce PBP foci in a system independent of *gdpP* to examine the cause-and-effect relationship between PBP foci and β-lactam resistance. It is possible that β-lactam resistance is caused by additional mechanisms. It is widely accepted that β-lactams cause cell wall damage by inducing imbalanced cell wall synthase and hydrolases activity (83). A groundbreaking study from *E. coli* shows that β-lactams trigger killing by inducing a toxic futile cycle of cell wall synthesis and recycling that depletes PG synthesis precursors (84). Changes in PG precursors and alteration of cell wall hydrolases have been reported in *gdpP* mutants (13, 52, 53). It remains to be further investigated whether changes in PG precursors and cell wall hydrolases contribute to β-lactam resistance or tolerance.

β-Lactam antibiotics target the transpeptidase activity of PBPs essential for PG cross-linking. In *S. aureus*, new PG synthesis occurs at the division septa, and all four native PBPs (PBP1–4) of *S. aureus* are known to localize at the septa during PG synthesis (72–74, 85). PBP1 is a high-molecular-weight (HMW) class B PBP with only transpeptidase activity, and it is essential for cell septation and separation (86, 87). PBP2 is a HMW class A PBP and is bifunctional with both transpeptidase and transglycosylase activities (88). It is also essential for cell division and remains localized at the division septum upon initiation stage of daughter cell separation (85). PBP3 and PBP4 are non-essential PBPs with only transpeptidase activity (78, 89, 90). Class B PBPs PBP1 and PBP3 form pairs with monofunctional transglycosylase FtsW and RodA (known as SEDS proteins), respectively, which participate in septal and sidewall PG synthesis (91). PBP4 is a class C PBP that is recruited to the division septum after PBP1 and PBP2 (92). It has carboxypeptidase activity and transpeptidase activity that contribute to the generation of highly cross-linked PG (93). Here, we show that all four PBPs aggregate into aberrant single foci in the *gdpP* mutant, and PBP2 foci were the most severe. Given their different functions, it is possible that individual PBPs may contribute differently to resistance. A transcriptomic analysis from an early study showed that among all *pbp* genes, only the expression of *pbp4* was increased by 2.71-fold in Δ*gdpP* cells (49). Another study reported that the amount of PBPs was not significantly changed in a *gdpP* mutant (15). Future work will be to dissect the contribution of each individual PBP.

How are PBPs mislocalized to single foci in the *gdpP* mutant? Our results showed that it is not *gdpP* itself, but elevated c-di-AMP level upon *gdpP* deletion or *dacA* overexpression dysregulates PBPs’ localization. It is not due to changes in PG cross-linking, as the PG cross-linking is relatively normal in Δ*gdpP* cells. Since all four PBPs are mislocalized and cell division is impaired in Δ*gdpP*, the mechanism might be more generic. As we did not observe mislocalization of ZapA, a cytoplasmic divisome protein, we speculate that the mislocalization of PBPs may be linked to changes in membrane structure. The Δ*gdpP* cells are deficient in cell division, which may indirectly affect PBP localization. In addition, various mechanisms have been reported to regulate the localization of PBPs in *S. aureus*, including the availability of transpeptidase substrates, wall teichoic acid synthesis, and proper assembly of divisome components (72, 85, 92, 94). However, unlike what has been shown in the literature, the foci formation of PBPs is a unique phenotype that has not been reported before. We suspect that there are unique mechanisms linking c-di-AMP accumulation and PBP localization, which will be studied further.

We show here that SpA is mislocalized in Δ*gdpP* cells, which is mediated by its YSIRK/G-S signal peptide. Surface proteins are important components of the staphylococcal cell envelope, which play crucial roles in staphylococcal pathogenesis (95). Surface proteins that carry a YSIRK/G-S signal peptide are known to be enriched at the septal membrane and deposited at the cross-wall, which allows efficient assembly of surface virulence proteins as PG synthesis mainly occurs at the septum (56). Our group recently published results from a comprehensive screening of *S. aureus* transposon mutant library (96). Similar to Δ*gdpP* but unlike Δ*ltaS,* all the mutants identified dysregulate the late-stage biogenesis of SpA (96). Defects in cell cycle and cell wall homeostasis are the shared mechanisms among these mutants (96). Here, in the Δ*gdpP* cells, we propose that SpA mislocalization is mainly due to cell division defect since the septal wall synthesis is not significantly altered. However, we cannot rule out that other mechanisms, such as increased autolysis in Δ*gdpP*, may play a role (13, 53). SpA is an important virulence factor of *S. aureus* that binds to host immunoglobulin and evades host immune response (97). We speculate that other YSIRK+ virulence proteins, such as ClfA and FnbpA, will show similar mislocalization as SpA, leading to virulence attenuation. It has been reported that Δ*gdpP* downregulates *spa* expression (49). Thus, while Δ*gdpP* is resistant to β-lactam antibiotics, it modulates the expression and assembly of surface virulence proteins. The exact mechanisms remain to be determined.

## MATERIALS AND METHODS

### Bacterial strains and growth conditions

Strains and plasmids used in this study are listed in Table S1. *E. coli* strain DC10B, DH5α, and DH5α λpir were grown in Luria–Bertani broth (LB) or on LB agar plates supplemented with the appropriate antibiotics. Ampicillin (Amp; 100 µg/mL) and trimethoprim (Tmp; 10 µg/mL) were used for plasmid selection in *E. coli. S. aureus* strains were grown in tryptic soy broth (TSB) or on tryptic soy agar (TSA) plates supplemented with the appropriate antibiotics: 7 µg/mL of chloramphenicol (Chl), 10 µg/mL of erythromycin (Ery), 10 µg/mL of trimethoprim (Tmp), and 25 µg/mL of tetracycline (Tet). ATc (200 ng/mL) was used to induce gene expression from the *P_tet_* promoter in pCL*itet*. 1% glucose and xylose were used to repress and induce *P_xyl_* promoter in the pTX plasmid. If not specified, overnight cultures were grown at 37°C with appropriate antibiotics and 1:100 refreshed to TSB the next day. Appropriate inducers were added to the refreshed cultures. Refreshed cultures were grown for 2–3 h to mid-log phase (OD_600_ = 0.8) and proceeded with different experiments.

### Construction of plasmids and strains

Primers used in this study are listed in Table S2. To construct Δ*gdpP*, an internal deletion of 1,529 bp of the *gdpP* gene (*SAOUHSC_00015*) including its SD sequence was deleted. The gene knockout vector pKOR1 plus cre-lox marker removal system was used to generate the *gdpP* mutant (64, 98). To generate pKOR1-*gdpP::erm-lox*, primers 1,143/1,144 were used to amplify the upstream DNA fragment*,* primers 1,147/1,148 were used to amplify *lox66-ermB-lox71* from *smc::erm-lox* (99), and primers 1,145/1,146 were used to amplify the downstream DNA fragment. The three fragments were digested by XhoI and PstI, ligated, and amplified by primers 1,143 and 1,148. The PCR product (up + erm + down) was digested by EcoRI and EcoRV and ligated to pKOR1. The resulting knockout plasmid pKOR1-*gdpP::erm-lox* was confirmed by restriction enzyme digestion and sequencing. The homologous recombination process followed the previous protocol plus *erm^+^* counter selection (98). The final deletion mutant of *gdpP* was confirmed by PCR and sequencing. SEJ1Δ*gdpP*, NewmanΔ*gdpP*, HG001Δ*gdpP*, and JE2Δ*gdpP* were constructed by transducing Δ*gdpP::erm* to WT strains. To remove the *erm^+^* marker, pRAB1 was transformed to SEJ1Δ*gdpP::erm* (64). The resulting SEJ1Δ*gdpP*::- was confirmed by PCR and sequencing. SEJ1Δ*pbp4* and SEJ1Δ*gdpP*Δ*pbp4* were generated by phage transduction of Δ*pbp4::Tn* from Nebraska Transposon Mutant Library (NTML) (100).

Mutagenesis PCR was used to construct pCL55-*sp(clfB)-spa* and pCL55-*sp(splE)-spa*. The DNA sequences encoding the signal peptides of *clfB* and *splE* were PCR-amplified from Newman using primers 424/425 and 562/563. The PCR products of the signal peptides were used as primers to mutate pCL55-*spa* (56), generating pCL55-*sp(clfB)-spa* and pCL55-*sp(splE)-spa*. To construct GFP-tagged PBP1–4, primers 702/648 were used to PCR amplify *pbp1* from SEJ1 genomic DNA. The PCR product was ligated with NheI/BglII digested pCL*itet-sfgfp-sfgfp* (63) via Gibson assembly (NEB), generating pCL*itet-sfgfp-pbp1*. Primers 704/650 were used to PCR amplify *pbp2* from SEJ1 genomic DNA. The PCR product was restricted by NheI/BamHI and ligated with plasmid pCL*itet-sfgfp-sfgfp* restricted by NheI/BglII, generating pCL*itet-sfgfp-pbp2*. Primers 705/652 were used to PCR amplify *pbp3* from SEJ1 genomic DNA. The PCR product was restricted by NheI/BamHI and ligated with plasmid pCL*itet-sfgfp-sfgfp* restricted by NheI/BglII, generating pCL*itet-sfgfp-pbp3*. The construction of pCL*itet-pbp4-sfgfp* has been described in our previous study (63). The *gdpP* complementation plasmid was generated as follows: primers 626/627 and 628/629 were utilized to amplify the *gdpP* operon promoter (290 bp upstream of SAOUHSC_00014) and the *gdpP* gene from SEJ1 genomic DNA. The PCR products of the *gdpP* promoter and *gdpP* gene were digested with NheI/SacI and SacI/BamHI respectively, and the pKK30 plasmid (101) was digested with NheI/BamHI. Digested products were ligated to generate pKK30-P*gdpP-gdpP*, simplified as pKK30*-gdpP*. For *dacA* overexpression, primers 1,149/1,150 were used to amplify *dacA* from RN4220 genomic DNA. The PCR product was digested with BamHI/SacI and ligated into the pTX plasmid carrying a xylose-induced promoter to generate pTX-*dacA*. To generate pALFS1-*ZapA-GFPmut2*, the *ZapA-GFPmut2* fusion was cut out from pJB67-*ZapA-GFPmut2* (70) by SalI/BamHI digestion and ligated with pALFS1, a modified pJB67 with the antibiotic *erm^R^* cassette replaced by *chl^R^*. The positive clones were confirmed by DNA sequencing and transformed into desired *S. aureus* strains.

### Immunofluorescence microscopy and quantification

SpA immunofluorescence microscopy was performed based on the protocols described in previous studies (56, 65–67). Briefly, in the cross-wall IF experiment, mid-log phase cultures were collected and treated with trypsin. Trypsinized cells were washed and incubated in fresh medium containing trypsin inhibitor for 20 min at 37°C to allow SpA regeneration. Subsequently, the cells were fixed and stained with SpA primary antibodies and fluorescently labeled secondary antibodies (anti-rabbit IgG conjugated to Alexa Fluor 488) (Invitrogen). In a membrane IF experiment, trypsinized cells were fixed and digested with lysostaphin (AMBI) on the glass slides for 2 min. The samples were fixed again in methanol and acetone, followed by immunostaining. All samples were stained in the dark with Hoechst 33342 DNA dye (Invitrogen) and Nile red (Sigma). Fluorescent images were captured by Nikon Scanning Confocal Microscope Eclipse Ti2-E with HC PL APO 63× oil objective (1.4 NA, WD 0.14 mm). For quantification of SpA localization, at least 300 cells for each sample from three independent experiments were analyzed with ImageJ (102). The total cell numbers with SpA signals and cells displaying cross-wall/septal SpA localization were counted. Unpaired *t*-test with Welch’s correction was performed for statistical analysis using GraphPad Prism: *P < 0.05; **P < 0.005; ***P < 0.0005; and ****P < 0.0001.

### Van-FL staining and cell cycle quantification

Overnight cultures were refreshed at 1:100 in TSB and incubated to OD_600_ of 1.0. The cells were resuspended in phosphate-buffered saline (PBS) (137 mM NaCl, 2.7 mM KCl, 10 mM Na_2_HPO_4_, and 1.8 mM KH_2_PO_4_, pH = 7.4), fixed with 1 mL 50% fixation solution (2.5% paraformaldehyde and 0.01% glutaraldehyde in PBS), washed twice with PBS, and then applied to poly-l-lysine-coated eight-well glass slides. Excess and non-adherent cells were washed away with PBS, and samples were then stained with 1 µg/mL BODIPY FL Vancomycin (Invitrogen) to stain the cell wall. Quantification of cell size and cell cycle: at least 300 cells per sample from three independent experiments were analyzed in ImageJ (102). The staphylococcal cell cycle has been previously defined (69): phase 1, cells without a septum; phase 2, cells with a partial septum; phase 3, cells with a complete septum and elongated shape. Unpaired *t*-test with Welch’s correction was performed for statistical analysis using GraphPad Prism.

### *dacA* overexpression

Bacterial cells with pTX-*dacA* were incubated in TSB overnight and subsequently washed twice with basic medium without glucose (10 g/L soy peptone, 5 g/L yeast extract, 5 g/L NaCl, and 1 g/L KH_2_PO_4_, pH = 7.2). Washed cells were inoculated 1:100 in 10 mL basic medium without glucose in a flask and were grown for 2 h to reach OD_600_ of 0.5. 1% xylose was added to induce *dacA* expression, while 1% glucose was added to repress its expression as a control. After an additional 2 h of incubation, the cells were harvested for further study.

### Intracellular c-di-AMP level measurement

Intracellular c-di-AMP levels were detected using a competitive enzyme-linked immunosorbent assay (ELISA) (51). Overnight bacterial cultures were diluted 1:100 in fresh medium, grown to mid-log phase, and normalized to OD_600_ of 1.0. The culture (2 mL) was harvested, washed, and resuspended in PBS. The cells were then mechanically lysed using a MiniBeadBeater-16 (BioSpec) with 0.1 mm silica beads at 3,450 RPM, 115 V for 30 s per cycle, for a total of eight cycles. Following lysis, silica beads were removed by centrifugation, and the resulting supernatant was collected as the cell lysate. Lysates were heated at 95°C for 10 min and diluted 1:20 for accurate measurement. C-di-AMP-specific ELISA kit (Cayman Chemical CYCLIC DI-AMP ELISA KT, 96SOLID) was utilized to determine the level of c-di-AMP, and the experiment was performed based on the manufacturer’s instructions. Briefly, 50 µL of the standard, sample, and control were added to each well of a 96-well plate pre-coated with antibody. Then, 50 µL of the HRP-linked cyclic di-AMP tracer solution was added to each well (except blank and total activity wells), followed by 50 µL of the c-di-AMP antibody (except NSB, blank, and total activity wells). The plate was incubated for 2 h at room temperature with gentle shaking. Then, the wells were washed and incubated with 175 µL TMB substrate for 30 min, followed by applying 75 µL stop solution. Absorbance was measured by a plate reader at 450 nm. Standard curves were determined with the c-di-AMP provided in the kit, and a four-parameter logistic curve was plotted. C-di-AMP level of each sample was determined by the standard curve. Three independent experiments were performed to calculate the c-di-AMP levels of each sample.

### Fluorescence microscopy of PBPs’ localization

For Bocillin-FL staining, bacterial cells at OD_600_ of 1.5 were harvested by centrifugation. The cell pellets were washed with PBS and incubated with 10 µM Bocillin-FL penicillin sodium salt (Invitrogen) in TSB at 37°C for 30 min. To study the localization of sfGFP-tagged PBPs, bacteria cells were incubated in TSB supplemented with 200 ng/mL ATc for 3 h to induce the expression of sfGFP-tagged fusion proteins. Subsequently, samples were harvested by centrifugation, washed, and resuspended in PBS.

Samples treated with penicillin G, vancomycin, oxacillin, and nafcillin were prepared as follows: SEJ1 and SEJ1Δ*gdpP* were treated with 0.01, 0.02, and 0.04 µg/mL penicillin G; 0.2 and 0.8 µg/mL oxacillin; 1 and 4 µg/mL nafcillin; and 0.25 and 0.5 µg/mL vancomycin. JE2 and JE2Δ*gdpP* were treated with 0.05, 0.1, 1, and 5 µg/mL penicillin G; 16 and 64 µg/mL oxacillin; 32 and 128 µg/mL nafcillin; and 0.25 and 0.5 µg/mL vancomycin. After 3 h of incubation, the cells were harvested, washed with PBS, and the OD_600_ was normalized to 1.5. The cells were then fixed in 1 mL of 50% fixation solution, washed twice with PBS, and applied to poly-l-lysine-coated eight-well glass slides (MP Biomedicals). Excess and non-adherent cells were washed away with PBS, and the samples were then stained with Hoechst 33342 DNA dye and Nile red or FM 4-64 Dye (Invitrogen). Samples were covered with 15 µL of SlowFade diamond antifade mountant (Invitrogen) and sealed with glass coverslips. All images were captured by Nikon Scanning Confocal Microscope ECLIPSE Ti2-E equipped with the HC PL APO 63× oil objective (1.4 NA, WD 0.14 mm).

For quantification and statistical analysis, at least 300 cells for each sample from three independent experiments were analyzed by ImageJ software (102). The total cell numbers and cells displaying Bocillin-FL foci or GFP foci were counted. Unpaired *t*-test with Welch’s correction was performed for statistical analysis using GraphPad Prism: *P < 0.05; **P < 0.005; ***P < 0.0005; and ****P < 0.0001.

### Fluorescent d-amino acid (FDAA) labeling

FDAA staining was performed as described earlier (63). Bacteria were cultured to mid-log phase and adjusted to OD_600_ of 0.8. The cells were then incubated with 500 µM HADA (TOCRIS) in TSB for 20 min at 37°C with shaking, followed by a single wash using 0.01 M PBS. Subsequently, the cells were incubated in TSB containing 500 µM RADA (TOCRIS) for another 20 min at 37°C, washed once with PBS, and then incubated in TSB with 500 µM OGDA (TOCRIS) for 20 min at 37°C, followed by washing with PBS. Washed cells were fixed with fixation solution for 20 min at room temperature, after which they were washed with PBS. Then, 50 µL of the resuspended cells was applied to poly-l-lysine coated glass slides and allowed to dry at room temperature. Samples were covered with 15 µL of SlowFade diamond antifade mountant and sealed with glass coverslips. Images were captured by Nikon Scanning Confocal Microscope ECLIPSE Ti2-E equipped with the HC PL APO 63× oil objective. For quantification of FDAA aberrant localization, at least 300 cells for each sample from three independent experiments were analyzed with ImageJ (102). The total cell numbers with FDAA aberrant foci formation and FDAA co-localization were counted separately. Unpaired *t*-test with Welch’s correction was performed for statistical analysis using GraphPad Prism: *P < 0.05; **P < 0.005; and ***P < 0.0005.

### Cell wall purification and HPLC analysis

Muropeptide purification from *S. aureus* was conducted according to established protocols with some modifications (103–105). Bacterial cells were incubated overnight and refreshed in 1 L of TSB with an initial OD_600_ of 0.1. The cells were harvested at OD_600_ of 0.6 and boiled in 5% SDS for 15 min to remove non-covalent bound proteins, followed by several washes with water to remove SDS. Staphylococcal cells were mechanically lysed by bead beater with 0.1 mm silica beads, beating 3,450 RPM at 115 V for 30 s with eight cycles (MiniBeadBeater-16, Biospec). Subsequently, silica beads were removed by centrifugation, and the cells were incubated with 10 µg/mL DNAse and 50 µg/mL RNAse at 37°C for 2 h with shaking. An overnight digestion with 50 µg/mL trypsin was performed to remove surface proteins. Then, the cells were treated with 48% HF for 48 h at 4°C to remove WTA, followed by washing several times with water to remove HF. Purified peptidoglycan was resuspended in Na-Phosphate buffer (50 mM Na_2_HPO_4_ and 50 mM NaH_2_PO_4_, adjusted to pH 7.0), normalized to an OD_600_ of 3.0, and treated with 50 U/100 µL mutanolysin (Millipore Sigma) overnight at 37°C to obtain muropeptides. Muropeptides were reduced by sodium borohydride in 500 mM sodium borate (pH = 9.0) for 20 min, and the reaction was quenched by adjusting the pH to 3.0 with 30% phosphoric acid. Then, samples were analyzed by reverse phase HPLC (Agilent 1260 Infinity II) using the InfinityLab Poroshell 120 EC-C18 (4.6 × 150 mm, 2.7 µm) HPLC column. Elution was performed using a gradient from 5% to 30% methanol in 100 mM Na-Phosphate buffer (0.4 mL/min) for 150 min and monitored at 205 nm. For quantification of PG profile, three independently grown cultures were utilized to purify the cell wall and analyzed by HPLC. The areas of muropeptide peaks were integrated and quantified using Agilent Data Analysis version 2.7 and presented as a percentage of the total.

### Antibiotic resistance and tolerance

Minimum inhibitory concentrations (MICs) were determined using the broth microdilution method according to CLSI standards (106). Overnight bacterial cultures were diluted 1:100 and grown to mid-log phase. 1 × 10^5^ colony-forming units (CFU) were inoculated into 0.2 mL of cation-adjusted Mueller-Hinton broth containing serial dilutions of antibiotic from 0.25 to 256.0 mg/L for penicillin G, oxacillin, nisin, vancomycin, and daptomycin, and from 0.25 to 2,048 mg/L for nafcillin. Cultures were incubated at 37°C for 48 h in a 96-well plate with shaking. The MIC was defined as the lowest antibiotic concentration with no bacterial growth. The MIC values were recorded at 24 and 48 h of incubation. The MIC measurement was reproduced from three independent experiments.

TDtest was performed to study the antibiotic tolerance (23, 80). Overnight bacterial cultures were diluted to an OD_600_ of 0.1, and 100 µL of the suspension was spread onto TSA plates. A 6-mm disk containing different amounts of penicillin G (0.5 µg for SEJ1 and 32 µg for JE2), oxacillin (2 µg for SEJ1 and 128 µg for JE2), nafcillin (16 µg for SEJ1 and 1,024 µg for JE2), and vancomycin (8 µg) was placed at the center of each plate, followed by incubation at 37°C for 18 h. After recording the inhibition zones, the antibiotic disks were replaced with disks containing 4 µg of glucose, and the plates were further incubated at 37°C for 48 h. Colonies that emerged within the inner half of the inhibition zone were defined as tolerant colonies. Data from three independent experiments were quantified, and unpaired *t*-test with Welch’s correction was performed using GraphPad Prism.

### Bocillin-FL binding measured by flow cytometry

Overnight bacterial cultures were diluted 1:100 in fresh medium, grown to mid-log phase, and normalized to OD_600_ = 1.0. 1 mL of bacteria cells were harvested, washed with PBS, and incubated with 100 nM Bocillin-FL penicillin sodium salt in TSB at 37°C for 30 min. The cells were washed twice with PBS and resuspended in PBS. Samples were analyzed on a BD LSR II Flow cytometer equipped with a 488-nm laser. A minimum of 1,000,000 events were collected per sample. FITC fluorescence intensity values were extracted from gated populations, background corrected using unstained controls, and a histogram showing FITC-A distribution among populations was generated by Floreada.io. Average FITC intensity values from three independent experiments were quantified, and unpaired *t*-test with Welch’s correction was performed using GraphPad Prism.

## Supplementary Material

Reviewer comments

## Data Availability

The data that support the findings of this study are available from the corresponding author upon request.

## SUPPLEMENTAL MATERIAL

Supplemental figures and tables spectrum.01430-26-s0001.docx Fig. S1 to S6, and Tables S1 and S2.

OPEN PEER REVIEW reviewer-comments.pdf An accounting of the reviewer comments and feedback.

ASM does not own the copyrights to Supplemental Material that may be linked to, or accessed through, an article. The authors have granted ASM a non-exclusive, world-wide license to publish the Supplemental Material files. Please contact the corresponding author directly for reuse.
