# Supplementary material for: Mislocalization of PBPs in Staphylococcus aureus gdpP mutant contributes to β-lactam resistance and surface protein cross-wall trafficking
Source: Microbiol Spectr. 2026 Aug 12;14(9):e01430-26. doi: 10.1128/spectrum.01430-26 (PMC13532199; doi:10.1128/spectrum.01430-26)
Supplement: Supplementary file 1 [file reviewer-comments.pdf]

ASM | Microbiology Spectrum

Mis-localization of PBPs in *Staphylococcus aureus gdpP* mutant contributes to β-lactam resistance and surface protein cross-wall trafficking

Yaosheng Jia, Ran Zhang, Salvatore Scaffidi, and Wenqi Yu

Corresponding Author(s): Wenqi Yu, University of South Florida

Review Timeline:

Submission Date:	May 23, 2026
Editorial Decision:	July 14, 2026
Revision Received:	July 22, 2026
Accepted:	July 24, 2026

Editor: Beiyan Nan

Reviewer(s): The reviewers have opted to remain anonymous.

Transaction Report:

(Note: With the exception of the correction of typographical or spelling errors that could be a source of ambiguity, letters and reports are not edited. The original formatting of letters and referee reports may not be reflected in this compilation.)

DOI: https://doi.org/10.1128/spectrum.01430-26

Re: Spectrum01430-26 (Mis-localization of PBPs in *Staphylococcus aureus gdpP* mutant contributes to β-lactam resistance and surface protein cross-wall trafficking)

Dear Dr. Wenqi Yu:

Thank you for the privilege of reviewing your work. I am pleased to inform you that your manuscript has been editorially accepted for publication. However, there are a few additional questions in the submission form that need to be answered before your manuscript is officially accepted. Once these are completed, please return your submission so that I can move your paper forward to acceptance.

Please return the manuscript within 60 days; if you cannot complete the submission questions within this time period, please contact me.

Thank you for submitting your paper to Spectrum.

Sincerely,
Beiyan Nan
Editor
Microbiology Spectrum

Dear Editor and Reviewers,

This manuscript was transferred from *Antimicrobial Agents and Chemotherapy* to *Microbiology Spectrum*. We thank the previous editor and reviewers for their time and effort reviewing our manuscript. In this resubmission, we have thoughtfully edited the manuscript in response to reviewers' comments and suggestions. Below is our point-to-point response to the reviewers' comments.

For ease of reading, our response to the reviewers' comments is marked in blue below. Modifications in the manuscript are marked in red in the marked-up manuscript file.

Point-to-Point Response to Reviewers' Comments

Reviewer #1 (Comments for the Author):

This manuscript investigates the consequences of gdpP deletion in Staphylococcus aureus, demonstrating aberrant mis-localization of penicillin-binding proteins (PBPs) from the division septum to the peripheral cell wall. The authors link this altered PBP organization to reduced surface protein deposition at the cross-wall, perturbations in cell division, and the emergence of β-lactam resistance through drug sequestration. Notably, the authors report that these strains do not develop vancomycin resistance, suggesting a mechanistically distinct phenomenon.

The study is clearly the result of substantial experimental effort and addresses an important question regarding the relationship between cyclic-di-AMP and antibiotic resistance. However, despite its potential significance, the manuscript suffers from conceptual ambiguities, insufficient quantitative analyses, and internal inconsistencies, which collectively dilute the impact of what could otherwise be a highly compelling study. In particular, the most intriguing observation - aberrant PBP foci formation and its role in ΔgdpP-mediated β-lactam resistance is not explored with sufficient rigor.

Below, I outline my major concerns, beginning with the central findings of the study and followed by issues that weaken the overall narrative.

1. PBP mis-localization, c-di-AMP, and β-lactam Resistance

The authors demonstrate that gdpP deletion results in PBP mis-localization, and they examine this phenomenon across multiple S. aureus strain backgrounds. However, the proposed link

between elevated c-di-AMP levels and PBP mis-localization is not convincingly established. Specifically:

• The percentage of cells exhibiting aberrant PBP foci is not reported in a quantitative or comparative manner across the mutant and wild-type strains.
• Whether the number and spatial distribution of PBP foci per cell correlate with the differing levels of β-lactam resistance observed among strains.

Moreover, while I recognize that elucidating the mechanistic origin of aberrant PBP foci formation may lie beyond the immediate scope of the study, the absence of a qualitative or hypothetical framework represents a missed opportunity. A more focused and stringent analysis of the c-di-AMP-PBP mis-localization-β-lactam resistance axis would substantially strengthen the manuscript and sharpen its central message.

We thank the reviewer for the comments and suggestions. All experiments presented in the manuscript have been performed at least three times, and all the data are quantified and analyzed with statistical analysis. In terms of the quantification and correlation among c-di-AMP, PBP foci and β-lactam resistance:

1) The quantification of cells exhibiting aberrant PBP foci are shown in Fig. 2B, 2C (Bocillin-FL foci), Fig. 4B (*dacA* overexpression) and Fig. 5B (individual PBP). The percentage of PBP foci is consistent in all *gdpP* mutants tested (65%-70% in *gdpP* mutants vs 2%-5% in WT strains as shown in Fig. 2BC).

2) The level of β-lactam resistance (4-8 fold increase in MIC) is also consistent in two *gdpP* mutants tested (Table 1).

3) The level of PBP foci formation correlates with c-di-AMP level: Δ*gdpP* accumulated higher amount of c-di-AMP (15–30-fold increase) and higher percentage of PBP foci (70%), whereas *dacA* overexpression accumulated lower amount of c-di-AMP (4–8-fold increase) and lower percentage of PBP foci (30%) (Fig. 2 and Fig. 4).

Thus, there is a strong correlation among c-di-AMP, PBP foci and β-lactam resistance. However, as also pointed out by Reviewer 2, the results are correlative, but not causative. We have revised the text throughout the manuscript to be more precise.

We agree that it will increase the impact of our study if we could figure out the mechanisms for PBP mis-localization. We tried to measure membrane fluidity, but the results are unclear. It will take us significant amounts of work to obtain data and to propose possible mechanism(s). As

the reviewer pointed out, it is out of the immediate scope of the current study. Per reviewer's suggestion, we have revised the discussion section to include possible mechanisms underlying PBP mis-localization.

2. Functional Activity of Mis-localized PBPs

The manuscript claims that mis-localized PBPs are inactive, yet this assertion is difficult to reconcile with the data presented, particularly in Figure 6, where clear FDAA labeling is evident. Importantly, Bocillin binding and FDAA incorporation measure distinct biological activities and cannot be directly compared to infer enzymatic inactivity. As currently presented, the data do not substantiate the claim that these PBPs are functionally inactive.

We thank the reviewer for the comments. Yes, most Δ*gdpP* cells showed normal FDAA staining and about 10%-25% cells showed aberrant FDAA foci formation (Fig. 6). In comparison, the rate of aberrant PBP foci formation is 65%-70% (Fig. 2). If the PBPs in these foci were fully active, they would incorporate FDAA locally to form FDAA foci; consequently, the FDAA foci rate would be close to 70%. However, this is not the case, which suggests that most PBPs in the foci are unable to incorporate FDAA. Thus, the results support the conclusion that PBPs in the foci are largely inactive. We are aware that Bocillin binding and FDAA incorporation measure different biological activities: the PBP foci formation did not reduce Bocillin-FL binding but failed to incorporate FDAA. Future work will be to investigate how aggregation of PBPs alters their activity. We apologize that the way we wrote was confusing. We have edited the text to clarify.

3. Effects on Spa Cross-wall Deposition and Cell Division

The assertion that PBP mis-localization disrupts Spa cross-wall deposition and early cell division events is, in my view, built on a weak experimental foundation. As a result, these interpretations risk diverting attention away from the manuscript's most impactful finding - namely, the relationship between aberrant PBP localization and β-lactam resistance.

We thank the reviewer for the thoughtful comments. We agree that there was conceptual inconsistency when we discussed the mechanisms for SpA mis-localization (please see our response to comment #4 below). We have revised the manuscript to clarify and included alternative mechanisms. We also agree that SpA results are a bit out of the axis of PBP localization and β-lactam resistance. However, we prefer to keep the results in the current manuscript for the following reasons: 1) surface proteins are key virulence factors widely found in gram-positive bacteria; the mechanisms of their cross-wall assembly are not fully understood yet. The results presented in the manuscript provide new insights into its mechanisms and

connection with cell envelope biology. 2) This part was inquired by a previous reviewer, and we have done extensive experiments to show that the mis-localization is YSIRK signal peptide dependent. 3) The SpA results are consistent with other result sections in the manuscript, but it was not well-thought out nor well-presented (please see our response to comment #4 below). We have revised the text now. 4) as the manuscript is now being submitted to *Microbiology Spectrum*, which publishes technically sound primary research, we prefer to keep the SpA results to be informative to the scientific community.

4. Vancomycin Resistance and Conceptual Inconsistencies

The discussion of vancomycin resistance is also conceptually problematic. If Spa mis-localization reflects a defect in peptidoglycan synthesis at the cross-wall, one might reasonably expect this to influence susceptibility to vancomycin, given its well-established mechanism of action. The apparent absence of a vancomycin phenotype, as presented, is not sufficiently reconciled with the proposed defects in septal cell wall organization. As a result, this section further weakens the overall coherence of the manuscript.

In summary, this work contains an important observation regarding PBP mis-localization and β-lactam resistance in S. aureus. However, insufficient quantitation, overextended interpretations, and unresolved conceptual inconsistencies currently limit its impact.

We thank the reviewer for the comments. The cross-wall incorporation of SpA requires proper cell cycle progression and septal cell wall synthesis. We think that SpA mis-localization is mainly caused by the cell cycle defect of the *gdpP* mutant, but not due to defects in septal wall synthesis, as the septal wall synthesis is relatively normal in the *gdpP* mutant (supported by muropeptide analysis shown in Fig. 7, vancomycin septal staining in Fig. 3A, and vancomycin susceptibility in Table 1, and FDAA staining in Fig. 6). In the wild-type staphylococcal cells, SpA precursors are secreted across both septal and peripheral membranes via SecA (1), primarily processed at the septal membrane via SpsB (2) and incorporated to septal lipid II by SrtA (3). However, SpA can be directly incorporated into peripheral mature wall via SrtA as (i) SrtA localizes at both septal and peripheral membrane in *S. aureus* (unpublished data) and (ii) our previous work showed that depletion of *spsB* and several other mutants impaired in cell cycle show SpA peripheral wall incorporation (2, 4, 5). In the wild-type cells, the majority of SpA is incorporated into septal peptidoglycan in the dividing cells. In the *gdpP* mutant, the cell division initiation is impaired, and most cells are not dividing (no septa). SpA is incorporated into peripheral peptidoglycan in these non-dividing cells, although the incorporation is less efficient and less SpA protein can be anchored to peripheral peptidoglycan since the anchoring sites

(uncross-linked glycine bridges) are limited in the mature peripheral peptidoglycan. When these non-dividing cells start to divide and form septa, SpA is incorporated into septal peptidoglycan. However, these cells have already accumulated SpA at the peripheral wall, which leads to SpA mis-localization phenotype: SpA appears at both septal and peripheral wall. The SpA phenotype does not conflict with the vancomycin results. Results of cell wall cross-linking analysis (Fig. 7), FDAA incorporation (Fig. 6) and vancomycin susceptibility (Table 1) are consistent, suggesting that the overall cell wall cross-linking and septal cell wall synthesis is relatively normal in the *gdpP* mutant. We agree that it was not well-thought out nor well-presented when we discussed the mechanisms for SpA mis-localization. We have revised the text to clarify.

Reviewer #2 (Comments for the Author):

This revised manuscript by Yaosheng Jia et al, presents a compelling, well-structured study with a clear mechanistic trajectory from phenotype to model, supported by multiple orthogonal approaches, including microscopy, biochemical assays, and phenotypic analyses. The distinction between resistance and tolerance is thoughtfully addressed as a significant strength of work. However, the current version relies heavily on correlative observations, and several key mechanistic conclusions are not yet fully supported by causal evidence. The central claim linking PBP foci formation to β-lactam resistance remains associative rather than definitive, and the proposed β-lactam sequestration model, while conceptually attractive, lacks direct experimental validation. Additional mechanistic gaps include the unresolved relationship between c-di-AMP accumulation, cell division defects, and PBP mis-localization, as well as limited resolution regarding the functional state of PBPs within foci. Furthermore, integrating the SpA trafficking phenotype with the antibiotic resistance framework requires clearer conceptual alignment. Finally, the writing overstates the strength of the conclusions relative to the available data. Overall, although this work is promising it could be significantly strengthened by clarifying the causal claims, key controls, and aligning the conclusions more closely with the data.

Reviewer critics

1. Lack of causal evidence linking PBP foci to β-lactam resistance. The manuscript establishes a strong correlation between PBP foci formation, elevated c-di-AMP, and increased β-lactam resistance. However, it does not yet demonstrate that PBP foci are causative rather than a parallel consequence of altered cellular physiology. The authors should consider introducing

perturbations that selectively disrupt foci (without fully restoring gdpP) and test whether resistance is reduced or conversely induce foci and assess whether resistance is observed.

We appreciate the reviewer's comments and suggestions. We have tried but have not been able to manipulate the PBP foci alone without having pleiotropic effects. We agree that the results obtained so far are correlative, but not causative. We have revised the manuscript to describe the results without making overstated claims.

2. The "β-lactam sequestration" model is not directly demonstrated.
The proposed model that peripheral PBPs sequester β-lactams is plausible but remains inferential. Increased Bocillin-FL binding alone cannot distinguish sequestration from altered accessibility, abundance, or membrane organization.
Could the authors provide evidence showing β-lactams preferentially accumulate in peripheral foci and septal PBPs at sublethal concentrations?

We thank the reviewer for the suggestion. We apologize for our inaccurate and misleading writing. The "β-lactam sequestration" model does not mean that peripheral PBP foci bind more β-lactam (we don't have data to support this claim). Rather, we meant that PBPs are mis-localized as peripheral foci, which deposit β-lactams to a wrong cellular location making them useless (shown in Fig. 2, 65%-70% Bocilin-FL foci in gdpP vs 2%-5% in WT). When we tested lower concentrations of Bocilin-FL, the percentage of Bocilin-FL foci formation was similar to the results shown in Fig. 2, while the Bocilin-FL signal intensity decreased at both septal and peripheral foci. We have deleted the inaccurate term "β-lactam sequestration" and replaced it with "mis-localize" or "mis-target" and revised the relevant text.

3. Unresolved mechanistic link between c-di-AMP, division defects, and PBP mislocalization.
While division defects (e.g., reduced ZapA rings) are documented, It is not established whether division defects drive PBP mislocalization, result from it, or occur in parallel.
The authors could clarify a pathway ordering through additional markers (e.g., FtsZ, divisome assembly) or perturbation-based experiments.

We agree that the relationship between cell division defects and PBP mis-localization remains unresolved. Based on the literature and our data, we propose the following:

1). It is known that cell division precedes cell wall synthesis in *S. aureus* (6). Divisome proteins FtsL/DivIB/DivIC recruit MurJ, the lipid II flippase, to initiate septal peptidoglycan synthesis (6). Depletion of *ftsZ* in *S. aureus* leads to enlarged cells, diminished septa formation and dispersed

PBP localization (7). The phenotypes of enlarged cell size and dispersed PBP localization upon *ftsZ* depletion are vastly different from PBP foci formation and smaller cell size of the *gdpP* mutant, suggesting that PBP foci formation is likely not caused by FtsZ inhibition.

2). Defects in cell wall synthesis can also inhibit cell division as seen in several other mutants we study, e.g. *ypfP, ltaA, tagO, mprF, dltC* (2, 4, 5). However, all these mutants have severe defects in cell wall structure and enlarged cells whereas the *gdpP* mutants have no significant change in cell wall cross-linking (although other structural change cannot be ruled out) and the cells are smaller. It suggests that the cell division defect of the *gdpP* mutant is likely not caused by cell wall synthesis defect.

As far as we are aware, the PBP foci formation in the *gdpP* mutant is a unique phenotype that has not been reported before, hinting that the underlying mechanisms are unique as well. Future work will be to investigate how c-di-AMP accumulation leads to cell division defects and PBP mis-localization, and whether/how these two phenotypes are connected. We agree it is an important aspect of the story. However, in our opinion, this part is beyond the immediate scope of the current study. Per reviewer's suggestion, we have revised the text and added discussion in the manuscript.

4. How are the authors distinguishing active versus inactive PBP populations? It is not clear.

We thank the reviewer for the inquiry. As shown in Fig. 6, most cells showed normal FDAA staining and about 10%-25% cells showed aberrant FDAA foci formation. In comparison, the rate of aberrant PBP foci formation is 70% (Fig. 2). If the PBPs in these foci were fully active, they would incorporate FDAA locally to form FDAA foci; consequently, the FDAA foci rate would be close to 70%. However, this is not the case, which suggests that most PBPs in the foci are unable to incorporate FDAA locally. We have edited the text to clarify.

5. Integration of SpA trafficking with antibiotic resistance is not fully resolved.
The SpA phenotype is compelling but appears only partially connected to the central β-lactam resistance model.
Integrate SpA into the mechanistic framework or present it as a secondary phenotype.

We thank the reviewer for the comments and suggestions. We agree that there was conceptual inconsistency when we discussed the mechanisms for SpA mis-localization. We also agree that SpA results are a bit out of the axis of PBP localization and β-lactam resistance. The cross-wall incorporation of SpA requires proper cell cycle progression and septal cell wall synthesis. We

think that SpA mis-localization is mainly caused by the cell cycle defect of the *gdpP* mutant, but not due to defects in septal wall synthesis, as the septal wall synthesis is relatively normal in the *gdpP* mutant (supported by muropeptide analysis shown in Fig. 7, vancomycin septal staining in Fig. 3A, and vancomycin susceptibility in Table 1, and FDAA staining in Fig. 6). In the wild-type staphylococcal cells, SpA precursors are secreted across both septal and peripheral membranes via SecA (1), primarily processed at the septal membrane via SpsB (2) and incorporated to septal lipid II by SrtA (3). However, SpA can be directly incorporated into peripheral mature wall via SrtA as (i) SrtA localizes at both septal and peripheral membrane in *S. aureus* (unpublished data) and (ii) our previous work showed that depletion of *spsB* and several other mutants impaired in cell cycle show SpA peripheral wall incorporation (2, 4, 5). In the wild-type cells, the majority of SpA is incorporated into septal peptidoglycan in the dividing cells. In the *gdpP* mutant, the cell division initiation is impaired, and most cells are not dividing (no septa). SpA is incorporated into peripheral peptidoglycan in these non-dividing cells, although the incorporation is less efficient and less SpA protein can be anchored to peripheral peptidoglycan since the anchoring sites (uncross-linked glycine bridges) are limited in the mature peripheral peptidoglycan. When these non-dividing cells start to divide and form septa, SpA is incorporated into septal peptidoglycan. However, these cells have already accumulated SpA at the peripheral wall, which leads to SpA mis-localization phenotype: SpA appears at both septal and peripheral wall. We prefer to keep the SpA results in the manuscript since SpA and other YSIRK+ proteins are key virulence factors of *S. aureus;* it is informative to report our data to the scientific community. Per reviewer's suggestion, we have revised the relevant text in the manuscript.

6. The mechanism underlying cell division delay remains unresolved.
As shown appears that are more phase 1 cells with reduced ZapA ring formation, both of which support a division-initiation defect. But the manuscript does not establish how elevated c-di-AMP causes that defect. Could the authors clarify, demonstrate: Is the division defect upstream of PBP mislocalization? Is PBP mis-localization upstream of the division defect? Are both parallel outcomes of altered osmotic/cell-envelope physiology?

We thank the reviewer for this inquiry. As we don't have direct experimental data, we can only speculate and discuss possible models (please see our response to comment #3). We agree that this part is very interesting and important. But we respectfully propose that this part is out of the immediate scope of the current manuscript.

Minor Weaknesses

7. Insufficient evidence supporting unchanged PBP abundance
The claim that PBP levels are unchanged is based on "data not shown, weakening the argument. Please, include supporting data, such as Bocillin-FL quantification or immunoblotting, in the supplement.

We have deleted this part since our results are too preliminary. In replacement, we added literature on PBP transcription and protein production.

8. Membrane contribution is not fully addressed
Membrane fluidity results are mixed and do not fully rule out membrane-driven effects. Could the authors clarify the limitations of the assay and moderate their conclusions regarding membrane independence?

We agree with the reviewer's comments. We have deleted this part since the results are unclear.

9. Limited quantitative support for growth and morphology differences
Growth defects and morphological changes are mentioned but not fully quantified in the main text. Inclusion of the growth curves and quantification of cell size and morphology, will be helpful.

The growth defect and small cell size have been well-documented in the literature (8). We think that these are not new results and not essential to be included in the current manuscript.

10. Lack of direct septal vs peripheral antibiotic targeting analysis
The central model would benefit from direct visualization of antibiotic distribution.
Include low-dose localization experiments or acknowledge this limitation.

We thank the reviewer for the suggestion. Please see our response to comments #2 above.

11. Overstatement of conclusions relative to data
Some language implies mechanistic certainty, even when only association is demonstrated. Use moderated phrasing to align with available evidence.

We have deleted "results interpretation" from the Results section to focus on objectively describing the results only. We have also modified the language in the Discussion section to be more accurate.

12. Manuscript organization and clarity

Section transitions and numbering are occasionally uneven, and some sections are text dense. Please, add conclusions to Results, and streamline the Introduction.

We have re-organized the result sections that were too long and clarified the conclusions.

13. Absence of a consolidated limitations section

Limitations are currently dispersed throughout the Discussion. Add a paragraph summarizing key limitations would be helpful.

We have revised the discussion part to make it more streamlined. However, we think that discussing the limitations for each major result is clearer for the logical flow.

References

1. Yu W, Missiakas D, Schneewind O. 2018. Septal secretion of protein A in *Staphylococcus aureus* requires SecA and lipoteichoic acid synthesis. Elife 7.
2. Zhang R, Jia Y, Scaffidi SJ, Madsen JJ, Yu W. 2025. Signal peptidase SpsB coordinates staphylococcal cell cycle, surface protein septal trafficking, and LTA synthesis. mBio 16:e0267324.
3. Perry AM, Ton-That H, Mazmanian SK, Schneewind O. 2002. Anchoring of surface proteins to the cell wall of *Staphylococcus aureus*. III. Lipid II is an in vivo peptidoglycan substrate for sortase-catalyzed surface protein anchoring. J Biol Chem 277:16241-8.
4. Scaffidi SJ, Zhang R, Jia Y, Yu W. 2026. Genome-wide screen identifies novel factors for surface protein cross-wall trafficking and cell envelope homeostasis in Staphylococcus aureus. J Bacteriol doi:10.1128/jb.00523-25:e0052325.
5. Zhang R, Shebes MA, Kho K, Scaffidi SJ, Meredith TC, Yu W. 2021. Spatial regulation of protein A in Staphylococcus aureus. Mol Microbiol 116:589-605.
6. Monteiro JM, Pereira AR, Reichmann NT, Saraiva BM, Fernandes PB, Veiga H, Tavares AC, Santos M, Ferreira MT, Macario V, VanNieuwenhze MS, Filipe SR, Pinho MG. 2018. Peptidoglycan synthesis drives an FtsZ-treadmilling-independent step of cytokinesis. Nature 554:528-532.
7. Pinho MG, Errington J. 2003. Dispersed mode of *Staphylococcus aureus* cell wall synthesis in the absence of the division machinery. Mol Microbiol 50:871-81.
8. Corrigan RM, Abbott JC, Burhenne H, Kaever V, Grundling A. 2011. c-di-AMP is a new second messenger in Staphylococcus aureus with a role in controlling cell size and envelope stress. PLoS Pathog 7:e1002217.

Re: Spectrum01430-26R1 (Mis-localization of PBPs in *Staphylococcus aureus gdpP* mutant contributes to β-lactam resistance and surface protein cross-wall trafficking)

Dear Dr. Wenqi Yu:

Your manuscript has been accepted, and I am forwarding it to the ASM production staff for publication. Your paper will first be checked to make sure all elements meet the technical requirements. ASM staff will contact you if anything needs to be revised before copyediting and production can begin. Otherwise, you will be notified when your proofs are ready to be viewed.

Data Availability: ASM policy requires that data be available to the public upon online posting of the article, so please verify all links to sequence records, if present, and make sure that each number retrieves the full record of the data. If a new accession number is not linked or a link is broken, provide production staff with the correct URL for the record. If the accession numbers for new data are not publicly accessible before the expected online posting of the article, publication may be delayed; please contact ASM production staff immediately with the expected release date.

Publication Fees: For information on publication fees and which article types have charges, please visit ourwebsite. We have partnered with Copyright Clearance Center (CCC) to collect author charges. If fees apply to your paper, you will receive a message from no-reply@copyright.com with further instructions. For questions related to paying charges through RightsLink, please contact CCC at ASM_Support@copyright.com or toll free at +1-877-622-5543. CCC makes every attempt to respond to all emails within 24 hours.

ASM Membership: Corresponding authors may join or renew ASM membership to obtain discounts on publication fees. Need to upgrade your membership level? Please contact Customer Service at Service@asmusa.org.

Embargo Policy: A press release may be issued as soon as the manuscript is posted on theSpectrum Latest Articles webpage. The corresponding author will receive an email with the subject line "ASM Journals Author Services Notification" when the article is available online.

Cover Image Submissions: If you would like to submit a potential Cover Image, please email a file and a short legend to spectrum@asmusa.org. Please note that we can only consider images that (i) the authors created or own and (ii) have not been previously published. By submitting, you agree that the image can be used under the same terms as the published article. Image File requirements: TIF/EPS, 7.5 inches wide by 8.25 inches tall (at least 2,250 pixels wide by 2,475 pixels tall), minimum 300 dpi resolution (600 dpi preferred), RGB, and no figure elements, e.g., arrows or panel labels. The legend should be a short description of the image, 1-2 sentences recommended. Please download and use this interactive template in Adobe the https://journals.asm.org/pb-assets/html/asm-cover-sizing-tool.html to ensure that your proposed cover image meets our size requirements (https://journals.asm.org/pb-assets/pdf-text-excel-files/ASM-Interactive-Sizing-Cover-Template-1715689791.pdf).

The ASM Journals program strives for constant improvement in our submission and publication process. Please tell us how we can improve your experience by taking this quick Author Survey.

Thank you for submitting your paper to Spectrum.

Sincerely,
Beiyan Nan
Editor
Microbiology Spectrum
