# Supplementary material for: Mislocalization of PBPs in Staphylococcus aureus gdpP mutant contributes to β-lactam resistance and surface protein cross-wall trafficking
Source: Microbiol Spectr. 2026 Aug 12;14(9):e01430-26. doi: 10.1128/spectrum.01430-26 (PMC13532199; doi:10.1128/spectrum.01430-26)
Supplement: Supplementary file 2 [file spectrum.01430-26-s0001.docx]

**Supplemental Materials**

**Fig. S1.** Construction of *S. aureus* Δ*gdpP*. The *gdpP* coding sequence, except for 449 bp at the 3’ end, was replaced by *ermB* cassette flanked with *lox* sites. *ermB* cassette was further removed by Cre recombinase. pKK30-P*_gdpP_-gdpP* plasmid was used to complement Δ*gdpP.*

**Fig. S2.** Deletion of *gdpP* in *S. aureus* Newman, HG001, and JE2 diminishes SpA cross-wall localization. (A) Immunofluorescence microscope images showing SpA cross-wall localization in WT and Δ*gdpP*. Nile red stained cytoplasmic membrane. Scale bar, 2 µm. (B) Quantification of SpA cross-wall localization from panel A. (C) Immunofluorescence images showing SpA septal membrane localization. (D) Quantification of SpA septal localization. Unpaired t-test with Welch’s correction was performed for statistical analysis: **P* < 0.05. Representative images and quantification are from three independent experiments.

**Fig. S3.** Bocillin-FL foci formation was growth-phase independent. SEJ1 and JE2 Δ*gdpP* cells were refreshed and grown for 4 h, 6 h, 8 h, and 24 h, and labeled with Bocillin-FL. Scale bar, 2 µm.

**Fig. S4.** Large image crops showing FDAA incorporation in SEJ1 and JE2 WT and Δ*gdpP* cells. Staphylococcal cells were sequentially incubated with three FDAAs: HADA (blue), RADA (red) and OGDA (green) and imaged by fluorescence microscopy. Scale bar, 4 µm.

**Fig. S5.** TDtest showing an increased β-lactam tolerance in SEJ1Δ*gdpP* but not in JE2Δ*gdpP.* (A) SEJ1 WT, Δ*gdpP*, and *gdpP* complementation strains were grown in disk with 0.5 µg penicillin, 2 µg oxacillin, 16 µg nafcillin, or 8 µg vancomycin for 18 hours, followed by changing disk containing 40% glucose and incubating for 48 hours. Representative images are from three independent experiments. (B) JE2 WT, Δ*gdpP*, and complementation strains growth in disk with 32 µg penicillin, 128 µg oxacillin, 1024 µg nafcillin, or 8 µg vancomycin for 18 hours, followed by changing disk containing 40% glucose and incubating for 48 hours. Representative images are from three independent experiments. (C) Enlarged figure from (A) SEJ1 Δ*gdpP* treated with penicillin, oxacillin, nafcillin, and colonies that emerged within the inner half of the inhibition zone were defined as tolerant colonies.

**Fig. S6.** PBP foci formation upon antibiotic treatment. (A) Bocillin-FL staining of SEJ1 (MSSA) WT and Δ*gdpP* treated with different concentrations of oxacillin (OXA) and nafcillin (NAF). (B) Bocillin-FL staining of JE2 (MRSA) WT and Δ*gdpP* treated with different concentrations of OXA and NAF. FM4-64 stains cytoplasmic membrane. Scale bar, 2 µm.

**Table S1. Strains and plasmids used in this study**

| **Strains or plasmids** | **Description^a^** | **Reference or source** |  |
| --- | --- | --- | --- |
| *E. coli* |  |  |  |
| DC10B | Cloning strain | (1) |  |
| DH5α | Cloning strain | NEB Gibson assembly kit |  |
| DH5αλpir | Cloning strain | (2) |  |
| *S. aureus* |  |  |  |
| SEJ1 | RN4220Δ*spa,* WYL112 | (3) |  |
| USA300 | JE2, WYL658 | NTML (4, 5) | |
| Newman | WYL001 | (6) | |
| HG001 | WYL564 | (7) | |
| WYL1207 | JE2Δ*gdpP::erm* | This study | |
| WYL1217 | SEJ1Δ*gdpP::erm* | This study | |
| WYL1209 | NewmanΔ*gdpP::erm* | This study | |
| WYL1211 | HG001Δ*gdpP::erm* | This study | |
| WYL1218 | JE2Δ*gdpP::erm* pKK30-*gdpP* | This study | |
| WYL1111 | SEJ1Δ*gdpP::erm* pKK30-*gdpP* | This study | |
| WYL1219 | NewmanΔ*gdpP::erm* pKK30-*gdpP* | This study | |
| WYL1220 | HG001Δ*gdpP::erm* pKK30-*gdpP* | This study |  |
| WYL480 | SEJ1 pCL*itet* | (8) |  |
| WYL478 | SEJ1 pCL*itet-spa,* Chl^R^, ATc-inducible | (8) |  |
| WYL709 | SEJ1Δ*gdpP::erm* pCL*itet-spa,* Chl^R^, ATc-inducible | This study |  |
| WYL1092 | SEJ1Δ*gdpP::erm* pKK30-*gdpP* pCL*itet-spa,* Chl^R^, ATc-inducible | This study |  |
| WYL743 | SEJ1, pCL55*-spa*, Chl^R^ | (8) |  |
| WYL1842 | SEJ1, pCL55-*sp(clfB)-spa*, Chl^R^ | This study |  |
| WYL1843 | SEJ1, pCL55-*sp(splE)-spa*, Chl^R^ | This study |  |
| WYL1844 | SEJ1Δ*gdpP::erm*, pCL55*-spa*, Chl^R^, Ery^R^ | This study |  |
| WYL1845 | SEJ1Δ*gdpP::erm*, pCL55-*sp(clfB)-spa*, Chl^R^, Ery^R^ | This study |  |
| WYL1846 | SEJ1Δ*gdpP::erm*, pCL55-*sp(splE)-spa*, Chl^R^, Ery^R^ | This study |  |
| WYL1847 | SEJ1Δ*gdpP::erm* pKK30-*gdpP* pCL55*-spa*, Chl^R^, Ery^R^, Tmp^R^ | This study |  |
| WYL1848 | SEJ1Δ*gdpP::erm* pKK30-*gdpP* pCL55-*sp(clfB)-spa*, Chl^R^, Ery^R^, Tmp^R^ | This study |  |
| WYL1849 | SEJ1Δ*gdpP::erm* pKK30-*gdpP* pCL55-*sp(splE)-spa*, Chl^R^, Ery^R^, Tmp^R^ | This study |  |
| WYL1854 | JE2, pCL55*-spa*, Chl^R^, ATc-inducible | This study |  |
| WYL1858 | Newman, pCL55*-spa*, Chl^R^, ATc-inducible | This study |  |
| WYL1856 | HG001, pCL55*-spa*, Chl^R^, ATc-inducible | This study |  |
| WYL1855 | JE2Δ*gdpP::erm*, pCL55*-spa*, Chl^R^, Ery^R^, ATc-inducible | This study |  |
| WYL1892 | NewmanΔ*gdpP::erm*, pCL55*-spa*, Chl^R^, Ery^R^, ATc-inducible | This study |  |
| WYL1857 | HG001Δ*gdpP::erm*, pCL55*-spa*, Chl^R^, Ery^R^, ATc-inducible | This study |  |
| WYL1307 | SEJ1 pCL*itet-sfgfp*, Chl^R^, ATc-inducible | This study |  |
| WYL1318 | SEJ1 pCL*itet-sfgfp-pbp1*, Chl^R^, ATc-inducible | This study |  |
| WYL1320 | SEJ1Δ*gdpP::erm* pCL*itet-sfgfp-pbp1*, Chl^R^, Ery^R^, ATc-inducible | This study |  |
| WYL1322 | SEJ1Δ*gdpP::erm* pKK30-*gdpP* pCL*itet-sfgfp-pbp1*, Chl^R^, Ery^R^, Tmp^R^, ATc-inducible | This study |  |
| WYL1324 | SEJ1 pCL*itet-sfgfp-pbp2*, Chl^R^, ATc-inducible | This study |  |
| WYL1326 | SEJ1Δ*gdpP::erm* pCL*itet-sfgfp-pbp2*, Chl^R^, Ery^R^, ATc-inducible | This study |  |
| WYL1328 | SEJ1Δ*gdpP::erm* pKK30-*gdpP* pCL*itet-sfgfp-pbp2*, Chl^R^, Ery^R^, Tmp^R^, ATc-inducible | This study |  |
| WYL1330 | SEJ1 pCL*itet-sfgfp-pbp*3, Chl^R^, ATc-inducible | This study |  |
| WYL1332 | SEJ1Δ*gdpP::erm* pCL*itet-sfgfp-pbp*3, Chl^R^, Ery^R^, ATc-inducible | This study |  |
| WYL1334 | SEJ1Δ*gdpP::erm* pKK30-*gdpP* pCL*itet-sfgfp-pbp*3, Chl^R^, Ery^R^, Tmp^R^, ATc-inducible | This study |  |
| WYL1100 | SEJ1 pCL*itet-pbp4-sfgfp*, Chl^R^, ATc-inducible | This study |  |
| WYL1102 | SEJ1Δ*gdpP::erm* pCL*itet-pbp4-sfgfp*, Chl^R^, Ery^R^, ATc-inducible | This study |  |
| WYL1115 | SEJ1Δ*gdpP::erm* pCL*itet-pbp4-sfgfp* pKK30-*gdpP*, Chl^R^, Ery^R^, Tmp^R^, ATc-inducible | This study |  |
| WYL1690 | SEJ1 pTX-*dacA*, Tet^R^, xylose-inducible | This study |  |
| WYL1692 | Newman pTX-*dacA*, Tet^R^, xylose-inducible | This study |  |
| WYL1694 | JE2 pTX-*dacA*, Tet^R^, xylose-inducible | This study |  |
| WYL1352 | SEJ1Δ*gdpP*::- | This study |  |
| WYL1356 | SEJ1Δ*gdpP::-*Δ*pbp4::erm*, Ery^R^ | This study |  |
| WYL1360 | SEJ1Δ*pbp4::erm*, Ery^R^ | This study |  |
| WYL1344 | SEJ1 pALFS1*-zapA-sfgfp*, Chl^R^, CdCl_2_-inducible | This study |  |
| WYL1346 | SEJ1Δ*gdpP::erm* pALFS1*-zapA-sfgfp*, Chl^R^, Ery^R^, CdCl_2_-inducible | This study |  |
| WYL1348 | SEJ1Δ*gdpP::erm* pKK30-*gdpP* pALFS1*-zapA-sfgfp*, Chl^R^, Ery^R^, Tmp^R^, CdCl_2_-inducible | This study |  |
| **Plasmids** |  |  |  |
| pCL*itet* | pCL55 containing ATc-inducible *P_tet_* promoter, Amp^R^ (*E. coli*), Chl^R^ (*S. aureus*), ATc-inducible | (3) |  |
| pCL*itet-spa* | Full length *spa* cloned in pCL*itet*, Amp^R^ (*E. coli*), Chl^R^ (*S. aureus*), ATc-inducible | (8) |  |
| pCL55-*spa* | Full length *spa* with its native core promoter cloned in pCL55, Amp^R^ (*E. coli*), Chl^R^ (*S. aureus*) | (9) |  |
| pCL55-*sp(clfB)-spa* | SpA signal peptide (encoding 1-36 aa of SpA) replaced by ClfB signal peptide in pCL55-*spa*, Amp^R^ (*E. coli*), Chl^R^ (*S. aureus*) | This study |  |
| pCL55-*sp(splE)-spa* | SpA signal peptide (encoding 1-36 aa of SpA) replaced by SplE signal peptide in pCL55-*spa*, Amp^R^ (*E. coli*), Chl^R^ (*S. aureus*) | This study |  |
| pKOR1-*gdpP*-erm | *gdpP* deletion vector | This study |  |
| pCL*itet-sfgfp-pbp1* | *sfgfp-pbp1* cloned in pCL*itet*, Amp^R^ (*E. coli*), Chl^R^ (*S. aureus*), ATc-inducible | This study |  |
| pCL*itet-sfgfp-pbp2* | *sfgfp-pbp2* cloned in pCL*itet*, Amp^R^ (*E. coli*), Chl^R^ (*S. aureus*), ATc-inducible | This study |  |
| pCL*itet-sfgfp-pbp3* | *sfgfp-pbp3* cloned in pCL*itet*, Amp^R^ (*E. coli*), Chl^R^ (*S. aureus*), ATc-inducible | This study |  |
| pCL*itet-pbp4-sfgfp* | *pbp4-sfgfp* cloned in pCL*itet*, Amp^R^ (*E. coli*), Chl^R^ (*S. aureus*), ATc-inducible | (10) |  |
| pKK30-*gdpP* | *gdpP* cloned in pKK30, Tmp^R^ (*E. coli*& *S. aureus*) | This study |  |
| pALFS1-*zapA-sfgfp* | *zapA-sfgfp* cloned in pALFS1, Chl^R^ (*S. aureus*), CdCl_2_-inducible | This study |  |
| pTX-*dacA* | *dacA* cloned in the pTX plasmid with xylose-induced promoter. Tet^R^ (*E. coli*& *S. aureus*) | This study |  |

^a.^Abbreviations: Chl, chloramphenicol; Ery, erythromycin; Amp, ampicillin; Tmp, trimethoprim; Tet, tetracycline; ATc, anhydrotetracycline.

**Table S2. Primers used in this study**

| **Primer No.** | **Sequence** | **Description** |
| --- | --- | --- |
| 424 clfB_F | tacatacagggggtattaatttgaaaaaaagaattgattatttgt | pCL55-*sp(clfB)-spa* |
| 425 clfB_R | tgagcttcatcgtgttgcgcagcttgtgcttgatgattgcct | pCL55-*sp(clfB)-spa* |
| 562 SplE_F | acatacagggggtattaatatgaataaaaatataatcatcaaaagt | pCL55-*sp(splE)-spa* |
| 563 SplE_R | tgagcttcatcgtgttgcgcagctttagccgtttgttgaataccct | pCL55-*sp(splE)-spa* |
| 702 pbp1_F | ggaggcgccgcaGCTAGCGGCGGAGGTGGCTCTgcgaagcaaaaaattaaaattaaaaa | pCL*itet-sfgfp-pbp1* |
| 648 pbp1_R | aacagatctttagtccgacttatccttgtcagt | pCL*itet-sfgfp-pbp1* |
| 704 pbp2_F | gcaGCTAGCGGCGGAGGTGGCTCTacggaaaacaaaggatcttctca | pCL*itet-sfgfp-pbp2* |
| 650 pbp2_R | gcgGGATCCttagttgaatatacctgttaatcca | pCL*itet-sfgfp-pbp2* |
| 705 pbp3_F | gcaGCTAGCGGCGGAGGTGGCTCTttaaaaagactaaaagaaaaatcaaatga | pCL*itet-sfgfp-pbp3* |
| 652 pbp3_R | gcgGGATCCttatttgtctttgtctttatttttatca | pCL*itet-sfgfp-pbp3* |
| 626 PgdpP_F | GGGGCTAGCtaatatatattttcaaagaatgaagcca | pKK30-P*gdpP-gdpP* |
| 627 PgdpP_R | GGGGAGCTCatttttaactatagctattattttaacct | pKK30-P*gdpP-gdpP* |
| 628 gdpP_F | GGGGAGCTCatagaggtggaataatgaatcggcagt | pKK30-P*gdpP-gdpP* |
| 629 gdpP_R | CCCGGATCCtcatgcatcttcactcctacttaattgt | pKK30-P*gdpP-gdpP* |
| 1143 gdpp up_F | ggggatatcggatccatagctatagttaaaaatatggg | pKOR1-*gdpP*-*erm* |
| 1144 gdpp up_R | gtactgcaggtcgacctattcactttttagaattatttttca | pKOR1-*gdpP*-*erm* |
| 1145 gdpp down_F | gtactcgaggtcgaccaagaacattcgatgcagcgagtta | pKOR1-*gdpP*-*erm* |
| 1146 gdpp down_R | acgaattccatttggtaaatccat | pKOR1-*gdpP*-*erm* |
| 1147 erm_F | taggtcgacctgcagtaccgttcgtataatgtatgctatacgaagttatagtattgtccgagagtga | pKOR1-*gdpP*-*erm* |
| 1148 erm_R | ttggtcgacctcgagtaccgttcgtatagcatacattatacgaagttatctccttggaag | pKOR1-*gdpP*-*erm* |
| 1149_dacA_F | tgtaggatccaggaggttctcgagatggatttttccaacttttttca | pTX-*dacA* |
| 1150_dacA_R | tgaattcgagctcttaattatttcacacctttcttttga | pTX-*dacA* |

**References**

1.	Monk IR, Shah IM, Xu M, Tan MW, Foster TJ. 2012. Transforming the untransformable: application of direct transformation to manipulate genetically Staphylococcus aureus and Staphylococcus epidermidis. mBio 3.

2.	Krute CN, Krausz KL, Markiewicz MA, Joyner JA, Pokhrel S, Hall PR, Bose JL. 2016. Generation of a Stable Plasmid for In Vitro and In Vivo Studies of Staphylococcus Species. Appl Environ Microbiol 82:6859-6869.

3.	Grundling A, Schneewind O. 2007. Genes required for glycolipid synthesis and lipoteichoic acid anchoring in Staphylococcus aureus. J Bacteriol 189:2521-30.

4.	Fey PD, Endres JL, Yajjala VK, Widhelm TJ, Boissy RJ, Bose JL, Bayles KW. 2013. A genetic resource for rapid and comprehensive phenotype screening of nonessential Staphylococcus aureus genes. mBio 4:e00537-12.

5.	Nuxoll AS, Halouska SM, Sadykov MR, Hanke ML, Bayles KW, Kielian T, Powers R, Fey PD. 2012. CcpA regulates arginine biosynthesis in Staphylococcus aureus through repression of proline catabolism. PLoS Pathog 8:e1003033.

6.	Duthie ES, Lorenz LL. 1952. Staphylococcal coagulase; mode of action and antigenicity. J Gen Microbiol 6:95-107.

7.	Herbert S, Ziebandt AK, Ohlsen K, Schafer T, Hecker M, Albrecht D, Novick R, Gotz F. 2010. Repair of global regulators in Staphylococcus aureus 8325 and comparative analysis with other clinical isolates. Infect Immun 78:2877-89.

8.	Zhang R, Shebes MA, Kho K, Scaffidi SJ, Meredith TC, Yu W. 2021. Spatial regulation of protein A in Staphylococcus aureus. Mol Microbiol 116:589-605.

9.	Yu W, Missiakas D, Schneewind O. 2018. Septal secretion of protein A in Staphylococcus aureus requires SecA and lipoteichoic acid synthesis. Elife 7.

10.	Zhang R, Jia Y, Scaffidi SJ, Madsen JJ, Yu W. 2025. Signal peptidase SpsB coordinates staphylococcal cell cycle, surface protein septal trafficking, and LTA synthesis. mBio doi:10.1128/mbio.02673-24:e0267324.
